# Antioxidant activity of *Flemingia praecox* and *Mucuna pruriens* and their implications for male fertility improvement

**DOI:** 10.1038/s41598-023-46705-9

**Published:** 2023-11-08

**Authors:** Shravan D. Kumbhare, Sanghadeep S. Ukey, Dayanand P. Gogle

**Affiliations:** 1 Post Graduate Teaching Department of Botany, RTM Nagpur University, Nagpur, 440033 India; 2 Department of Botany, Lokmanya Tilak College, Yavatmal, 445304 India; 3 Post Graduate Teaching Department of Molecular Biology and Genetic Engineering, RTM Nagpur University, Nagpur, 440033 India

**Keywords:** Biochemistry, Chemistry

## Abstract

Globally, 15–24% couples are unable to conceive naturally and 50% of cases of this problem are due to infertility in males. Of this, about 50% of male infertility problems are developed due to unknown reasons called as idiopathic infertility. It is well established that, reactive oxygen species (ROS) have negative impact on male fertility and are involved in 80% of total idiopathic male infertility cases. Medicinal plants are considered as an alternative approach for mitigating the health problems. The plants with good antioxidant capacity can improve the male infertility symptoms generated by ROS. Such medicinal plants can be used to alleviate the symptoms of male infertility with their diverse phytoconstituents. *Mucuna pruriens* is a well-accepted herb, with its seeds being used to improve the male fertility in various ways and one of the ways is by eliminating the ROS. In our field survey, another plant, *Flemingia praecox,* although less known, its roots are used in all problems related to the male fertility by tribal people of the Gadchiroli district of Maharashtra, India. The study was conducted to determine in vitro antioxidant potential of *F. praecox* and compared the results with the well-established male fertility improving plant *M. pruriens* with special emphasis on medicinally important roots of *F. praecox* and seeds of *M. pruriens.* The objective of the study was investigated by studying their total phenol (TPC) and flavonoid (TFC) content, antioxidant parameters (DPPH, FRAP, ABTS, DMPD, β-carotene bleaching and TAA) and finally DNA damage protection capacity of the plant extracts was studied. The plant parts used for the medicinal purposes have been investigated along with other major parts (leaves, stem and roots of both the plants) and compared with synthetic antioxidants, BHA, BHT and ascorbic acid. Moreover, the inhibition of two male infertility enzyme markers, PDE5 and arginase by *F. praecox* root and *M. pruriens* seed extract was also studied in vitro. The results showed that *F. praecox* possesses higher antioxidant activity than *M. pruriens* in the majority of studies as observed in TFC, DPPH, TAA, ABTS and DMPD assays. However, *M. pruriens* seeds showed best results in TPC, FRAP and DNA damage protection assay. *F. praecox* root extract also gave better PDE5 inhibition value than *M. pruriens* seeds. This study will help to establish the authenticity of *F. praecox* used by tribal people and will encourage its further use in managing the male infertility problems.

## Introduction

It is evident from previous large scale surveys that sperm count had declined by 50–60% globally during the last 60-year^1–3^. Male infertility is associated with greater incidence of cancer^4^, obesity, diabetes^5^, metabolic syndrome^6^ and also with mortality and can even cause problems in the health of future progeny^5^. Therefore, in a greater perspective, male infertility should not be seen only as a medical condition affecting fertility, but also general health and wellbeing^7^. The problem of male infertility is heterogeneous in origin which may be the consequence of genetic or environmental factors or both. The genetic factor includes, microdeletions in Y-chromosome, autosomal deletions, X-linked gene copy number variations, mutation in Cystic Fibrosis Transmembrane Conductance Regulator (CFTR) gene, defects in DNA repair mechanisms, etc.^8^. Other factors contributing to this problem includes environmental or occupational exposure to toxicants, lifestyle like smoking, alcohol consumption, drugs, psychological stress^9^ and recreational drugs which acts at the level of hypothalamic–pituitary–gonadal axis or directly on spermatogenesis consequently causing infertility^10^. However, in most cases the causes of male subfertility are poorly understood or not known, called idiopathic male infertility^11^. Recently, it has been reported that the epigenetic modifications, like abnormal DNA methylation, small non-coding RNA, histone tail modification^8^, single nucleotide polymorphism^12^, etc. in reproduction-related genes are responsible factors for idiopathic male infertility. However, many of these genetic problems are linked with antioxidant or ROS genes^12^ which might influences the critical balance between antioxidant and ROS.

ROS are one of the most closely associated factors involved in deciding the male infertility. ROS or the free radicals (FR) are the molecules with at least one unpaired electron. It is generated as a result of oxygen metabolism. The unpaired electrons make ROS a highly reactive and damaging chemical^13^. Low levels of ROS are required in various events of fertilization^13^ however, its excessive production, because of any reason and if it is not counterbalanced by body’s own antioxidant defences like superoxide dismutase, catalase, glutathione peroxidase and glutathione, etc.^14^ then it will lead to oxidative stress (OS). Consequently, it will cause oxidative damage to spermatozoa by increasing lipid peroxidation in its plasma membrane and thus alter the sperm functioning^15^. About 80% of the idiopathic infertile male^16,17^ and 30–40% of males with known causes has been reported to have elevated levels of ROS called as male oxidative stress infertility (MOSI)^18^. This oxidative stress will results in the protein, lipid and DNA damage in and around sperm atmosphere resulting in decline of fertility. The good thing about OS is that it can be reversed by using oral antioxidants and thus provides a good opportunity for treatment^9^.

**Figure 1:**
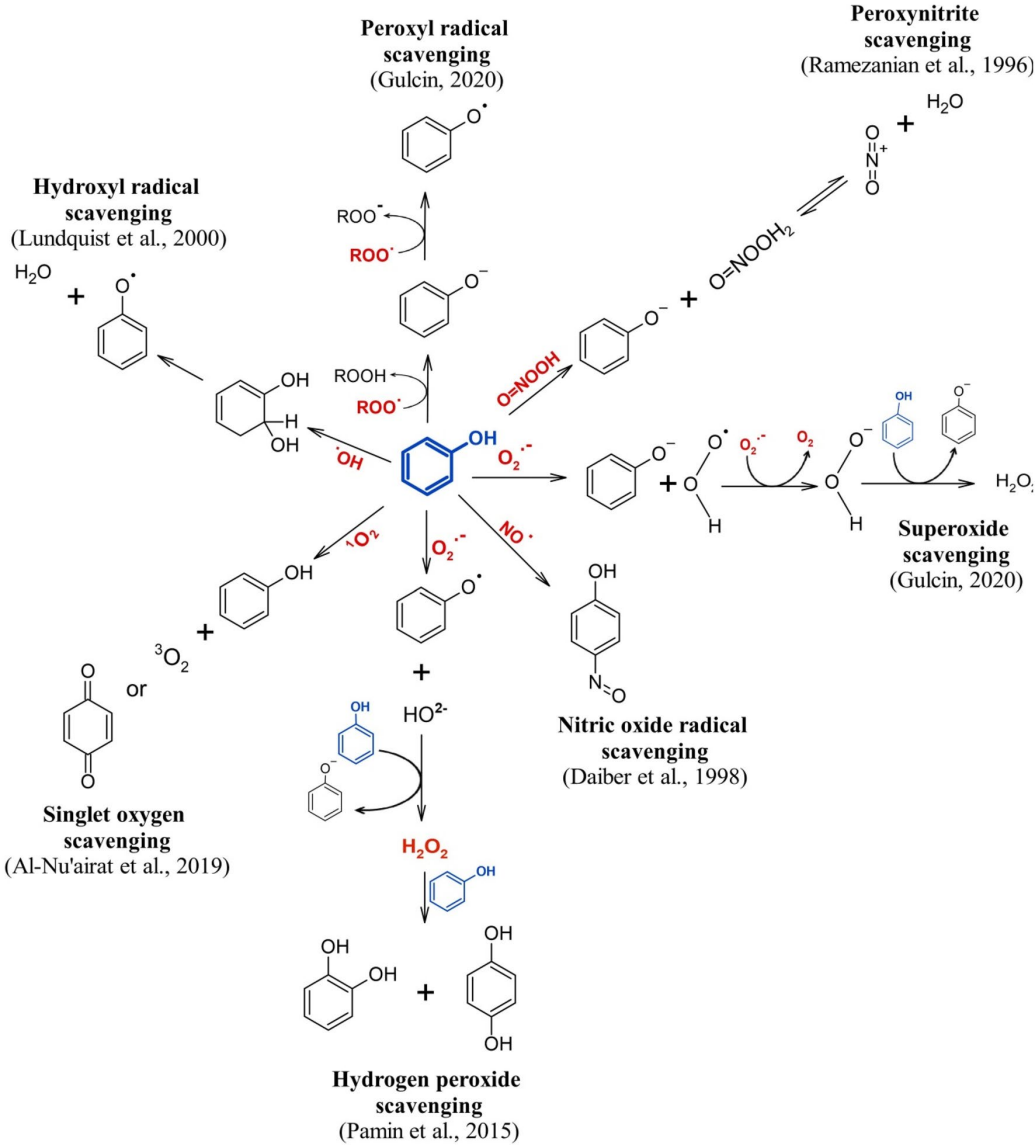
Phenol (blue) with its different mechanism of action against ROS (red).

**Figure 2:**
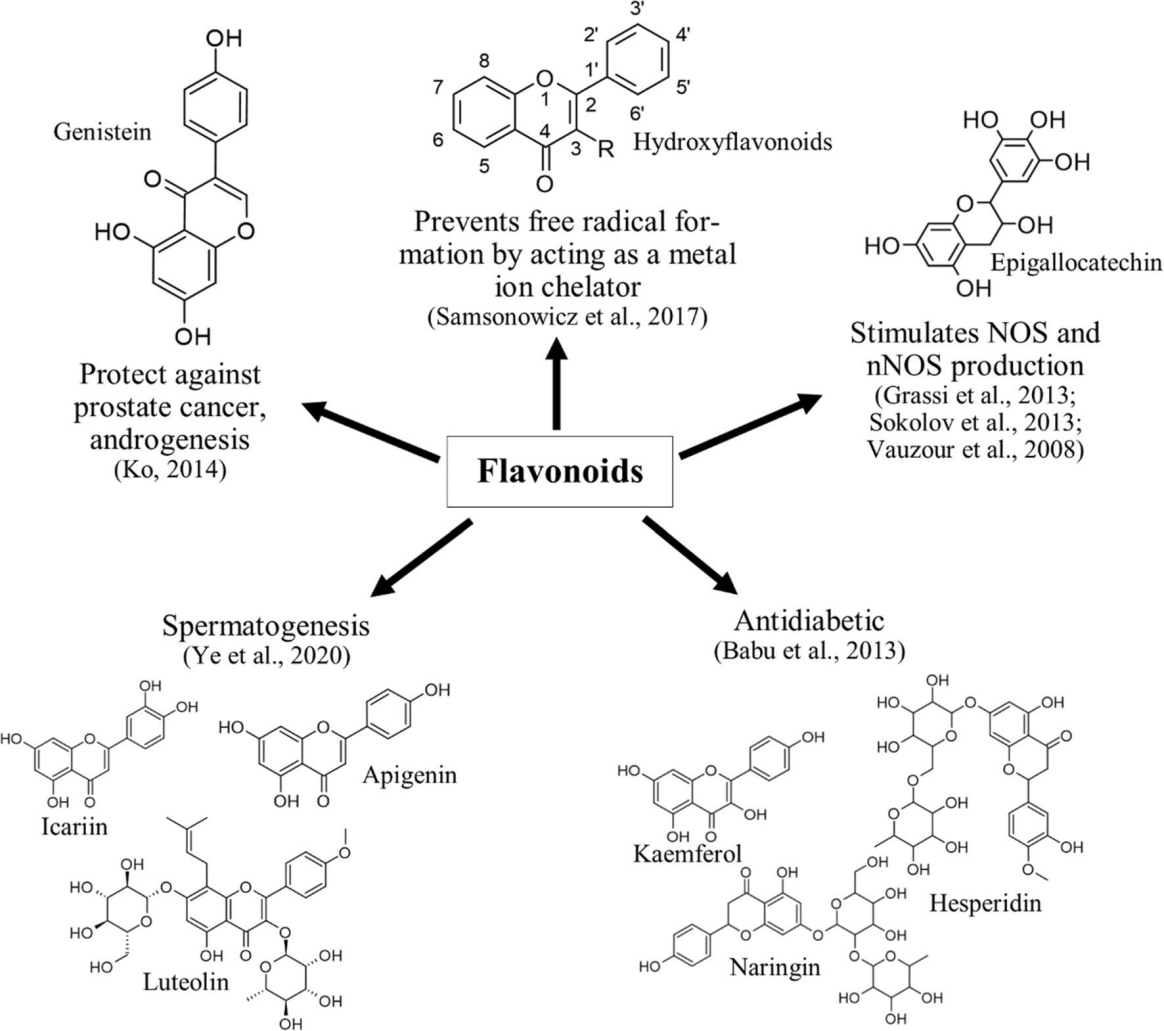
Flavonoids contribute in improvement of male fertility by different mechanisms.

The plant based antioxidants can become a good alternative to mitigate the problem of MOSI. The major antioxidant compounds in the plants are phenolics and flavonoids which work by eliminating and preventing the production of ROS^19^. Phenolic compounds have potential to scavenge major ROS and FR by different ways (Fig. 1). Moreover, the chemical structure of phenolics is more crucial than their concentration as it determines the extent of their absorption in the plasma^20^. Flavonoids, in the same line, although having the better antioxidant potential than phenols^21^ but due to their low absorption through intestinal route it was thought to work in improving the male fertility in different ways. It is evident that flavonoids improve male fertility preferably by modulating the cell signalling pathways and improving^22–26^ (Fig. 2). Hence, the plants with high concentration of different phenolic compounds including flavonoids can be used as a good source of antioxidants to alleviate the problem of male infertility.

*M. pruriens* is a well-recognized plant and traditionally been used for improving the male fertility. It improves male fertility by reducing ROS level, restoring mitochondrial membrane potential, regulating apoptosis^27^, controlling unspecific generation of ROS^28^, reactivating the antioxidant defense system, managing stress^29^, reducing lipid peroxidation^30^, acting on hypothalamus–pituitary–gonadal (HPG) axis and increasing levels of hormones^28^. Moreover, it is also thought that the male specific hormone production is assisted by presence of an active component, levodopa in its seeds^28^. However, a study showed that, apart from levodopa, other more superior bioactive components must be present in its seeds^31^.

Genus *Flemingia* consists of 44 species and two varieties that are mostly distributed in old world tropics^32^. In India, it is represented by 27 species and one variety^33^. The genus *Flemingia* is not only known for its high concentration of flavonoids but also contains its good diversity including flavones, flavanones, isoflavones, isoflavanone, chalcones, dihydrochalcones, flavonols, santalin flavones, flavanol, chromone and diflavone^34^. Traditionally, genus *Flemingia* have been used in the treatment of diseases like epilepsy, insomnia, ulcer, pain, swelling and regardless of a long traditional use of some species, this genus has not been explored properly^35^. However, the selected taxon for the study, *F. praecox* var. *robusta* (*F. praecox* hereafter) is endemic to India and has been reported in various parts of India^36,37^ and its phytochemical study was not done before. Interestingly, the traditional medicinal practitioners in Gadchiroli district of Maharashtra, India use this plant against male infertility problems. Therefore, we hypothesizing that *F. praecox* must be having chemical properties specific for improving the male fertility.

To check this hypothesis, we have conducted in vitro antioxidant studies on both *F. praecox* (its leaves, stem and medicinally important roots) and the well-recognized and traditionally used plant *M. pruriens* (its roots, leaves, stem and medicinally important seeds) under the similar analytical conditions and compared the findings. The correlational studies were also performed to discuss probable action mechanisms of these plants on ROS. Finally, inhibition of two male infertility markers, Phosphodiesterase 5 (PDE5) and arginase by the plant extract were also studied in vitro.

Based on recent literature reviews, it was observed that among various *Flemingia* species recognized for their medicinal properties, the most important organ with medicinal use was its roots^38–47^ followed by leaves and stems^35,48^. A very few studies have demonstrated the use of its seeds for medicinal purposes^35^. Moreover, none of the work has shown its any organ with capacity to ameliorate the male reproductive health. Furthermore, the traditional medicinal practitioners of Gadchiroli district were also denied the use of its seeds in male infertility cases. Due to these reasons, and most importantly, the extremely limited availability, we have not included the seeds of *F. praecox* in our studies.

## Results and discussion

### Preliminary phytochemical availability test

We have tested the availability of phenols, flavonoids, glycosides, alkaloids, terpenoids, tannins, steroids and saponins (Table 1). These tests were performed because we did not find any previous studies in literature on phytochemistry of *F. praecox.* The results obtained were compared with the* M. pruriens.* Multiple tests were performed for various categories of secondary metabolites. All parts of *F. praecox* have shown positive results in all the tests performed for phenols, flavonoids and also to some extent tannins. However, terpenoids and glycosides are almost absent in the *F. praecox* extracts which is also the case with the *M. pruriens* extract. *M. pruriens* also showed presence of phenols and flavonoids except its seed and root which showed negative results in some of the tests. Alkaloids were present in the leaves of both the plants. The roots of *F. praecox* and leaves of *M. pruriens* also give positive results for the presence of steroids. *M. pruriens* seeds also possessed good foaming capacity indicating the presence of saponins.

**Table 1:** Phytochemical availability tests in major plant parts of *F. praecox* and *M.* pruriens; presence of the phytochemical is indicated by ‘ + ’ and its absence indicated by ‘–’.

*F. praecox*				*M. pruriens*			
Plant parts	Leaf	Stem	Root	Leaf	Stem	Root	Seed
Phenols							
Ferric chloride test	+	+	+	+	+	–	+
Flavonoids							
F1	+	+	+	+	+	+	+
F2	+	+	+	+	+	–	–
F3	+	+	+	+	+	+	–
Alkaloids							
A1	+	–	–	+	–	–	+
A2	+	–	–	+	–	–	–
A3	+	+	–	+	+	+	+
A4	+	–	–	+	–	–	–
Steroids							
Salkowski test	–	–	+	+	–	–	–
Tannins							
T1	+	+	+	+	+	+	+
T2	+	+	+	+	+	+	+
T3	+	+	+	+	+	+	+
T4	–	+	+	–	+	–	+
Saponins							
S1	+	+	–	–	–	–	+
S2	–	–	–	–	–	–	+
Glycosides							
G1	–	–	–	–	–	–	–
G2	–	–	+	–	–	–	+
G3	–	–	–	–	–	–	–
Terpenoids							
Ter1	–	–	–	–	–	–	–
Ter2	–	–	–	–	–	–	–

A1: Hager’s test, A2: Dragendorff’s test, A3: Mayer’s Test, A4: Wagner’s test, F1: Lead acetate test, F2: Shinoda test, F3: Alkaline reagent test, G1: Keller-kiliani test, G2: Legal’s test, G3: Liebermann’s test, S1: Foam test, S2: Olive oil test, T1: Bramer’s Test, T2: Lead acetate test, T3: Potassium dichromate test, T4: Gelatin Test, Ter1: Acetic anhydride test, Ter2: Chloroform test.

Alkaloids are produced by the plants mainly for deterring herbivory by vertebrates which are also observed to have a negative role in pharmacological context^49^. Except phenolic and flavonoid glycosides all other glycosides are known to have adverse effects on^50,51^. Hence, the absence or low levels of glycosides and alkaloids in medicinally important plant parts i.e. seeds of *M. pruriens* and roots of *F. praecox* eliminates its possible side effects on the health. Previous data indicated the absence of alkaloids in aqueous and methanolic extract of *M. pruriens* leaf however, in our analysis all the tests performed showed its presence. Moreover, the terpenoids were absent in our analysis but they were found by other workers^52^. Previously alkaloids were isolated from *M. pruriens* leaves^53^ and seeds^54^ and its bioactivity was also studied which validates our positive results for alkaloids in *M. pruriens*^55^. Our results of saponins, tannins and flavonoids in *M. pruriens* were in accordance with results obtained by previous workers^52^. Steroids were reported in *M. pruriens* seeds^56^ however, in our analysis it was not detected in seeds but observed in the leaf.

Work has been done on various species of *Flemingia* like *F.*^57–59^*, F. macrophyla*^60^*, F. chappar*^61^*, F. philippinensis*^62^*, F. faginea*^48^*, F. grahmiana*^63^* F. stricta*^64^, etc. but no phytochemical work has been found in the literature on the species *F. praecox*. However, these species have shown the presence of phenols, flavonoids, steroids, tannins, glycosides, alkaloids and saponins and in most of the species the terpenoids were not^64,65^ our preliminary analysis also, the terpenoids were not detected in *F. praecox*.

### Quantification of phenolics

#### In the plant parts

**Figure 3:**
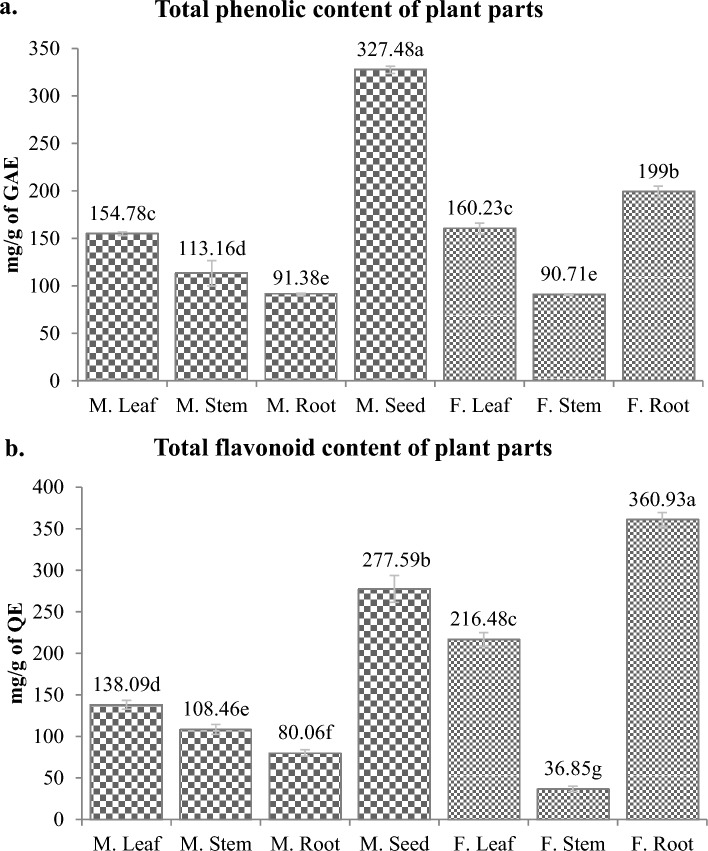
Total phenolics in methanolic extract of organs of *M. pruriens* (M.) and *F. praecox* (F.) (**a**) and total flavonoids in methanolic extract of parts of *M. pruriens* and *F. praecox* (**b**)*.* Values are presented as means of three readings ± SD (standard deviation). Highest to lowest values are shown in alphabetical order. Means with the different letter are significantly different at 95% confidence interval (*p* < *0.05*) according to Tukey’s multiple range test.

**Figure 4:**
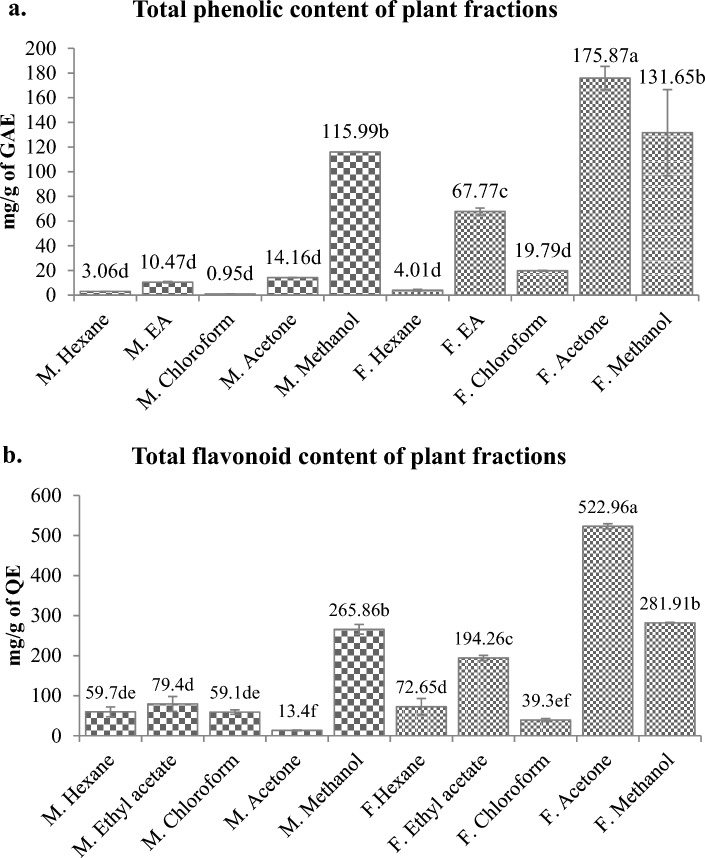
Total phenolics (**a**) and flavonoids (**b**) in different fractions made by sequential extraction of *M. pruriens* seeds (M.) and *F. praecox* roots (F.) in different solvents. Different letters represent significant differences at the *p* < *0.05* level.

Phenols and flavonoids are major groups of secondary metabolites that are known to have maximum shares in total antioxidant potential of any plant^19^. TPC and TFC were calculated first in all the plant parts (Fig. 3a and b) and then the plant parts that gave the highest values were fractionated by using various solvents with increasing polarity and again TPCs and TFCs of these fractions were estimated (Fig. 4a and b).

In our analysis, the significantly highest phenolic content was observed in *M. pruriens* seeds which is 327.48 ± 3.81 mg of gallic acid equivalent per gram of plant extract (mg GAE/g) followed by *F. praecox* roots containing 199.00 ± 5.96 mg GAE/g. However, leaves of both the plants showed statistically similar concentrations of phenol that is 154.78 ± 1.98 and 160.23 ± 5.85 mg GAE/g in *F. praecox* and *M. pruriens* respectively. The roots of *M. pruriens* and stem of *F. praecox* contain its minimum concentration (91.38 ± 0.88 and 90.71 ± 0.77 mg GAE/g respectively).

Unlike phenols, the highest level of flavonoids was estimated in *F. praecox* root that is 360.93 ± 8.49 mg of quercetin equivalent per gram of extract (mg QE/g) followed by *M. pruriens* seeds containing 277.59 ± 16.14 mg QE/g. *F. praecox* leaves also have shown the better levels of flavonoid (216.48 ± 8.49 mg QE/g) than other remaining plant parts. *F. praecox* stems contain lowest flavonoid content among the rest of the plant parts of both the plants (36.85 ± 3.21 mg QE/g).

#### In the fractions

On the basis of results obtained, we have selected *M. pruriens* seeds and *F. praecox* roots which are also the parts that are being used for improving the male fertility and attempted to quantify the phenols and flavonoids from their serial fractions made in different solvents with increasing polarity (Fig. 4a and b). The serial fractionation was done in the following sequence, n-hexane → ethyl acetate → chloroform → acetone → methanol. In our study, acetone extracted the highest fraction of phenol from *F. praecox* roots (175.87 ± 9.536 mg GAE/g). Methanol also dissolved significantly higher phenols from *F. praecox* roots (131.65** ± **34.90 mg GAE/g) than the remaining solvents. Also ethyl acetate fraction of *F. praecox* root has shown good phenolic value (67.77** ± **2.830 mg GAE/g). Among the *M. pruriens* seed fractions, the highest phenolic value was obtained in its methanolic fraction (115.99 ± 0.270 mg GAE/g) which is also statistically similar to phenols obtained in the methanolic fraction of *F. praecox* root.

The TFC analysis of different solvent fraction showed that *F. praecox* root contain significantly higher flavonoids that are soluble in acetone (522.96 ± 6.55 mg QE/g) followed by methanolic fraction (281.914 ± 1.07 mg QE/g). The ethyl acetate also extracted considerable flavonoids from *F. praecox* roots (194.26 ± 6.68 mg QE/g). Among *M. pruriens*, its methanolic fraction contains a maximum flavonoid than other fractions (265.86 ± 12.05 mg QE/g) indicating methanol as the best solvent for flavonoid extraction from *M. pruriens*.

Previously, the phenol content of *M. pruriens* was quantified by using different extraction solvents and methods. Due to its medicinal property, phenols were quantified mostly from seed extracts made in water, ethanol and methanol and it was found in the range of 3.9 to 230 mg GAE/g^66–70^, clearly showing that solvent influences the phenolic extraction. These levels were much lower than our quantified results in crude methanolic seed extract. Some studies however showed a considerably high level of phenolics up to 3730 mg GAE/g of extract^71^.

As stated earlier, the species *F. praecox* was not studied in the context of its phytochemistry. This may be due to its very low population size or its rarity in nature. However, we studied its phytochemistry for the first time from its restored population in our experimental field. In literature, we found that most of the work was done on *F. philippinensis*^72^. In its leaves, the phenols were 40 mg GAE/g and the roots showed 49 mg GAE/g. Other species of *Flemingia* expectedly showed varied amounts of phenols which ranges from 12 mg GAE/g in *F. strobilifera and F. vestita* to 280 mg GAE/g in *F. faginea*^47,48,57,73,74^ In our studies on *F. praecox* the phenols were found in good concentration i.e. 199 mg GAE/g in roots and also its leaves contain considerably higher phenols than in the leaves of *F. philippinensis*^72^*.* Thus our observation indicates that *F. praecox* can be the better source of phenolic antioxidants among its other species.

The methanolic extract of *M. pruriens* seeds has significantly higher concentration of phenols than its other organs which may be the reason for using its seeds for improving the male fertility. On the other hand, methanolic extract of *F. praecox* roots also showed considerably higher levels of phenols than its other organs which here as well can be the reason for its use in alleviating the male infertility problems by conventional medicinal practitioners. Moreover, acetone and methanol fractions of *F. praecox* give significantly higher phenol estimates even better than the similar fractions of *M. pruriens* seeds. Thus it can be concluded that *F. praecox* and *M. pruriens* are both relatable in the context of its phenolic content and medicinal property.

Previous studies have reported the high levels of flavonoids and also new flavonoids have been discovered from time to time from different species of *Flemingia.* Moreover, in vitro and preclinical properties of these flavonoids have also been reported by various researchers^42,75–79^. Some researchers also worked on isolation and in vitro properties of new flavonoids from leaves of other *F. praecox*^63,80,81^.

In *M. pruriens*, previous studies indicated its flavonoids are in the range of 63 to 807 mg QE/g of aqueous extract^70,71^ and 423 mg QE/g of ethanolic extract^82^ again indicating the place of origin of the plant and extraction procedure affecting the quantified values. In our analysis we observed maximum flavonoids in the *M. pruriens* seeds than its other organs studied which again signifying the use of its seeds for the medicinal purpose.

Studies on the flavonoids in different species of *Flemingia* showed that its value was ranging from 0.75 to 52.76 mg rutin equivalent per g aqueous^47,73^ and from 7.69 to 30.58 mg QE/g of hydro-alcoholic^74,83^. In one more study on the *F. faginea* leafy shoot found to contain 33.31 mg QE/g flavonoids in its aqueous extract. However, our study on *F. praecox* showed significantly high concentration of flavonoids in both its roots and leaf. Moreover, the analysis of flavonoid content in different solvent fractions of its roots shown even higher flavonoids specially in acetone fraction followed by methanolic fraction, indicating the acetone as a better solvent for flavonoid isolation from *F. praecox* roots which is also specified earlier by Pisoschi et al.^84^ Comparison of flavonoids in *M. pruriens* and *F. praecox* indicates that *F. praecox* species under study is far better source of flavonoids than its counterpart *M. pruriens*. *F. praecox* contain at least about more than two folds of flavonoid concentration in its acetone fraction than any fractions of *M. pruriens* and about 30% more in its medicinally important organ root than *M. pruriens* seeds. Even the leaves of *F. praecox* contain considerably good concentration of flavonoids.

Flavonoids are important group of secondary metabolite consist of cyclized diphenylpropane structure^19^ and are secreted in plants in the form of pigments in flowers, fruits, seeds, and leaves for recruiting pollinators and seed dispersers, in defence as feeding deterrent and antimicrobial agents, and in UV protection^85^. However, due to its diverse structure it is also found to have important medicinal applications. It was observed that in contrast to simple phenols which have mainly antioxidant properties (Fig. 1)^22,86^. Flavonoids show antioxidant activity mostly by chelating free radical forming metal ions like Fe^2+^ by formation of coordinate bonds with them by its –C=O and –OH groups^87^. Along with its major property of modulation of cell signalling pathway it was reported that flavonoids have capacity to improve vascular endothelial function by increasing the production of nitric oxide (NO) through endothelial nitric oxide synthase (eNOS)^88^. Flavonoids also have a neuroprotective role as it stimulates neuronal nitric oxide synthase (nNOS)^89,90^ and also possesses antidiabetic properties due to its insulin production capacity therefore improving the diabetes mediated vascular dysfunction^91^. Moreover, another group of flavonoids, isoflavones, have prostate cancer inhibition capacity by hormone dependent signalling pathway^92^ along with various spermatogenesis promoting effects^93^ (Fig. 2). This male fertility improving role of flavonoids can be met by its high levels in the *F. praecox.*

### Phenol and flavonoid detection in plant fractions by HPLC–MS/MS analysis

In *F. praecox* root, higher number of phenolic compounds were detected compared to the seeds of *M. pruriens* (Tables 2 and 3). Many of these compounds have been reported to have a positive impact on male fertility through various mechanisms. Some of these compounds possess antioxidant properties and may contribute to the reduction of oxidative stress^21^ or they are acting at different levels of male reproductive system.

**Table 2:** Phenolic compounds detected in *F. praecox* root methanolic fraction by HRLC-MS/MS analysis.

Sr. no	Compound name	CID	m/z	Phenolic class
1	Epigallocatechin	72,277	307.0799	Flavonoids
2	Daidzein	5,281,708	255.0647	Isoflavonoids
3	N1, N5, N10-tricoumaroyl spermidine	14,777,879	584.2731	Cinnamic acids and derivatives
4	Hellicoside	5,281,778	657.194	Cinnamic acids and derivatives
5	Xanthohumol	639,665	355.1528	Linear 1,3-diarylpropanoids
6	Licocoumarin A	5,324,358	407.1838	Isoflavonoids
7	Glycyrrhizaisoflavone B	10,546,844	367.1163	Isoflavonoids
8	4ʹ-O-methylkanzonol W	131,751,269	351.1211	Isoflavonoids
9	Licoisoflavone A	5,281,789	355.1164	Isoflavonoids
10	Kanzonol K	131,753,069	437.1938	Isoflavonoids
11	Kanzonol L	131,753,032	489.2245	Isoflavonoids
12	Isoliquiritigenin	638,278	257.0798	Linear 1,3-diarylpropanoids
13	Curcumin II	5,469,424	367.1524	Diarylheptanoids
14	Osajin	95,168	405.1677	Isoflavonoids
15	Kuwanon Z	21,594,954	593.1443	Flavonoids
16	Lespenefril	5,486,199	577.15	Flavonoids
17	2-Methyl-5-(8-pentadecenyl)-1,3-benzenediol	6,452,209	331.2592	Phenols
18	Metaxalone	15,459	222.112	Phenol ethers
19	2″,4″,6″-triacetylglycitin	131,751,611	595.143	Isoflavonoids
20	Camellianin A	5,487,343	643.1785	Flavonoids
21	Mulberranol	71,438,979	439.1742	Flavonoids
22	Kanzonol Z	10,319,154	407.1842	Flavonoids
23	N1,N5,N10-tris-trans-p-coumaroylspermine	10,908,386	641.3444	Cinnamic acids and derivatives
24	Kuwanone G	5,281,667	693.231	Flavonoids
25	2,2-dimethyl-3,4-bis(4-methoxyphenyl)-2H-1-benzopyran-7-ol acetate	255,270	431.1776	Isoflavonoids
26	Procyanidin B7	13,990,893	579.1482	Flavonoids
27	Ononin	442,813	431.1327	Isoflavonoids
28	Chrysin	5,281,607	255.0645	Flavonoids
29	Rutin	5,280,805	609.1403	Flavonoids
30	Isorhamnetin 3-glucoside 4′-rhamnoside	44,259,360	623.1558	Flavonoids
31	[Gallocatechin(4alpha- > 8)] 2catechin	14,890,508	897.2087	Flavonoids
32	Catechin-(4alpha- > 8)-gallocatechin-(4alpha- > 8)-catechin	131,752,348	881.2134	Flavonoids
33	Iriomoteolide 1a	16,723,501	505.3181	Phenylpropanoids

**Table 3:** Phenolic compounds detected in *M. pruriens* root methanolic fraction by HRLC-MS/MS analysis.

Sr. no	Compound name	CID	m/z	Phenolic class
1	(Z)-N-feruloyl-5-hydroxyanthranilic acid	10,087,955	330.097	Cinnamic acids and derivatives
2	MS 3	100,450	411.141	Phenylpropanoids
3	Senkirkine	5,281,752	366.19	Phenylpropanoids
4	Dipivefrin	3105	352.213	Phenol esters
5	5-(3′,4′,5′-trihydroxyphenyl)-gamma-valerolactone	44,389,277	223.061	Phenols
6	2,6-Dihydroxyphenylacetate	440,944	167.035	Flavonoids
7	Beclomethasone dipropionate	21,700	563.235	Flavonoids
8	10-Acetoxyligustroside	102,117,098	641.211	Flavonoids
9	Apimaysin	194,566	559.147	Flavonoids
10	Luteolin	5,280,445	285.041	Isoflavonoids
11	LysoPE(18:2(9Z,12Z)/0:0)	52,925,130	476.282	Phenylpropanoids

For instance, the epigallocatechin and other catechins from *F. praecox* have been shown to reverse testicular damage^94^ possibly due to their active antioxidant^95^ or DNA damage protection properties^96^. Similar positive effects on male fertility have been reported for compounds such as Lespenefril^24^, Chrysin^97^ and rutin^98^, all found in *F. praecox*. Spermidine derivatives like N1, N5, N10-Tricoumaroyl spermidine have been associated with ameliorative effects on sperm disorders in diabetic mice^99^. Other compounds in *F. praecox*, including Ononin^100^, Procyanidin B7, Curcumin II^101^ and Mulberranol^102^ have spermatogenic effect by modulating testosterone and other sex hormone levels. Moreover, flavonoids like Isoliquiritigenin have been reported to ameliorate sexual dysfunction^103^, while Licocoumarin A has been identified as an estrogen modulator^104^. Lastly, Xanthohumol has demonstrated the capacity to inhibit the growth and invasion of prostate cancer cells^105^.

On the other hand, in *M. pruriens* seed extract, the reported phenolic compound 5-(3′,4′,5′-Trihydroxyphenyl)-gamma-valerolactone which has been reported to possess neuroprotective properties^106^ and thus might have implication in the psychogenic male infertility. Another compound, the isoflavonoid, Luteolin have a well-known positive role in the process related to steroidogenesis, apoptosis and in stress response^107^. However, Beclomethasone dipropionate another compound detected in the *M. pruriens* seeds has been associated with negative effects on the reproductive function of male rats^108^. These findings collectively suggest that the presence of these phenolic and flavonoid compounds in both *F. praecox* roots and *M. pruriens* seeds may contribute to the improvement of male fertility, although through different mechanisms and at various levels within the male reproductive system.

### In vitro antioxidant capacity

The high concentrations of phenols and flavonoids in the medicinally used *M. pruriens* seeds and *F. praecox* roots also indicated the possibility of having high antioxidant values. Antioxidants are a major primary defence system against ROS and FR. It is well established from previous research that ROS and FR are the important contributory factors in various diseases including male infertility^17,109–111^. To check this hypothesis we studied the antioxidant properties of the plant parts of *M. pruriens* and *F. praecox* by DPPH (2,2-diphenyl-1-picrylhydrazyl), ABTS (2,2′-azino-bis-(3-ethylbenzothiazoline-6-sulfonic) acid) and DMPD (N, N-dimethyl-p-phenylenediamine) free radical scavenging assay, β-carotene bleaching, FRAP (Ferric ion reducing antioxidant power) and phosphomolybdenum antioxidant assay. The results obtained were compared with artificial antioxidants, butylated hydroxyanisole (BHA), butylated hydroxytoluene (BHT) and ascorbic acid.

#### DPPH⋅ scavenging activity

**Figure 5:**
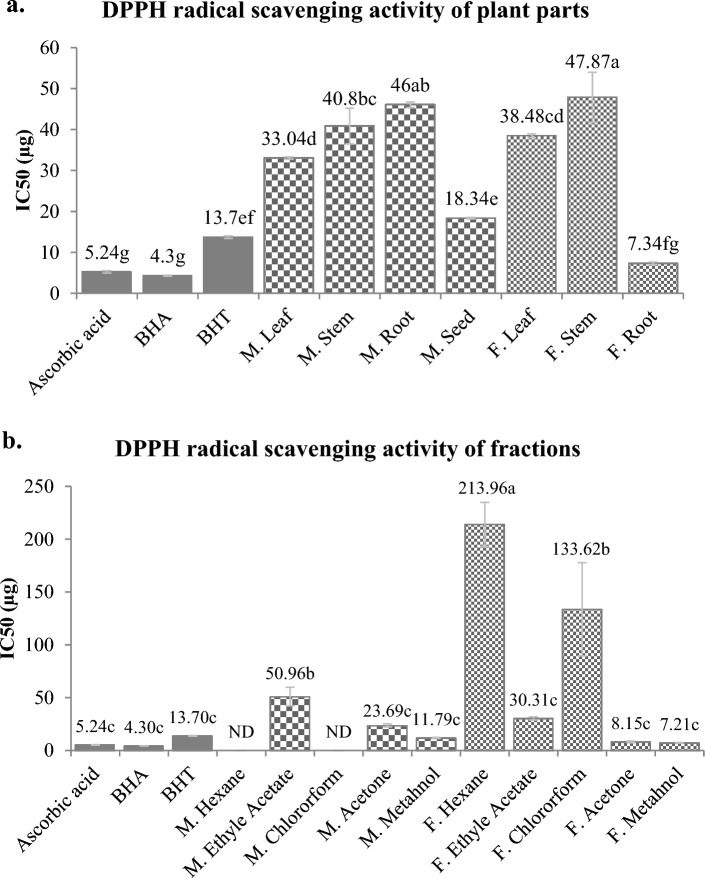
DPPH radical scavenging capacity of plant parts of *M. pruriens* and F*. praecox* (**a**) and the sequentially extracted fractions (**b**) of *M. pruriens* seeds and F*. praecox* roots. Different letters represent significant differences at the *p* < *0.05* level. *ND* not detected.

The DPPH radical scavenging activity is based on the reduction of purple coloured DPPH⋅ to its yellow hydrazine product (DPPH-H) by hydrogen or electron donating capacity of the plant compounds^19,112^. We have studied DPPH⋅ scavenging activity of plant parts (Fig. 5a) and the different fractions of *M. pruriens* seeds and *F. praecox* roots (Fig. 5b). The study revealed that *F. praecox* root has best scavenging activity among all the plant parts with IC_50_ (half-maximal inhibitory concentration) value 7.34 ± 0.315 µg and is also statistically similar in its scavenging activity to artificial antioxidants, ascorbic acid (5.24 ± 0.29 µg) and BHA (4.3 ± 0.12 µg) and even better than BHT (13.7 ± 0.31 µg). Other workers studied DPPH⋅ scavenging activity in different species of *Flemingia*, mostly in its roots^47,48,57,74,83^ and leaves^63^. In leaves of *F. grahmiana,* Gumula et al. showed IC_50_ value 5.9 µg whereas others studies on roots of various species of *Flemingia*, the IC_50_ for DPPH⋅ scavenging was ranged from best in *F. faginea* (15.04 µg)^48^ to the least in *F. vestita* (287 µg)^74^. However, among *M. pruriens*, its seed possesses the highest scavenging activity with IC_50_ value 18.34 ± 0.182 µg which is statistically similar to BHT. In previous studies on alcoholic and hydro-alcoholic extract of *M. pruriens* seeds, the DPPH⋅ scavenging activity in terms of IC_50_, was in range of 5.1 to 61.02µg^67–70^. Among the fractions of *M. pruriens* seed and *F. praecox* root, best DPPH⋅ scavenging activity was observed in methanol and acetone fractions of *F. praecox* root with IC_50_ values 7.21 ± 0.26 µg and 8.15 ± 0.83 µg respectively followed by methanol fraction of *M. pruriens* seeds having IC_50_ value 11.79 ± 0.51 µg. These all values are statistically similar to standards used at *p* < *0.05*. Hexane and chloroform fraction of *M. pruriens* seeds did not show any scavenging activity at the used concentration.

#### ABTS^⋅+^ scavenging activity

**Figure 6:**
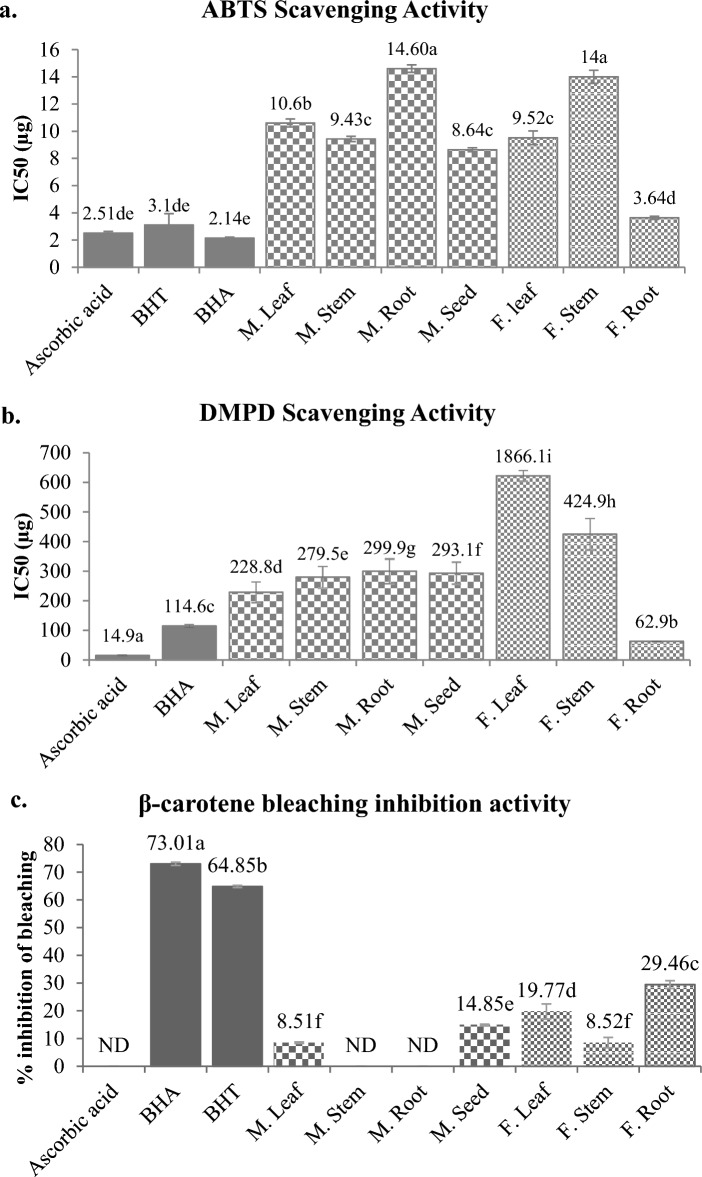
ABTS (**a**), DMPD (**b**) radical scavenging and β-carotene bleaching inhibition activity (**c**) of plant parts of *M. pruriens* and F*. praecox.* Different letters represent significant differences at the *p* < *0.05* level. *ND* not detected.

ABTS^⋅+^ scavenging activity demonstrates the capacity of the phytochemicals to neutralize ROS by hydrogen atom transfer (HAT) or single electron transfer (SET) mechanism^19^. The best HAT or SET capacity again shown by *F. praecox* roots having ABTS^•+^ scavenging IC_50_ value 3.63 ± 0.112 µg (Fig. 6a). Statistical tests shows that ABTS^•+^ scavenging potential of *F. praecox* root is statistically similar (*p* < *0.05*) to ascorbic acid (2.50 ± 0.125 µg) and BHT (3.10 ± 0.832 µg). The artificial antioxidant, BHA have shown the best ABTS^•+^ scavenging activity with IC_50_ value 2.14 ± 0.066 µg. Previous work on other species of *Flemingia* like *F. faginia*^48^ and *F. vestita*^74^ found the ABTS^⋅+^ scavenging IC_50_ value 67.33 µg and 11.49 µg respectively. These activities shown by other species are much lower than our studied species *F. praecox*. In the case of *M. pruriens*, seeds (8.64 ± 0.149 µg) and stem (9.43 ± 0.196 µg) have shown better scavenging activity than its other organs but significantly lower than its counterpart *F. praecox* root. At the end, *M. pruriens* root (14.60 ± 0.273 µg) and *F. praecox* stem (14.00 ± 0.482 µg) have shown lowest capacity to scavenge ABTS radicals. However, a large range of ABTS^⋅+^ scavenging values were observed by other workers in *M. pruriens* which is 6.009 µg as found by Njemuwa et al.^69^ to 137 µg which was observed by Chittasupho et al.^70^.

#### DMPD^⋅+^ scavenging

Another radical scavenging activity investigated with DMPD radical cation (DMPD^•+^) which is based on the HAT and SET mechanism of radical scavenging. This assay is less sensitive to hydrophobic and more specific for the hydrophilic antioxidants. This is opposite to DPPH⋅ and ABTS^⋅+^ scavenging assay^19^. The best scavenging results were again observed in *F. praecox* roots (Fig. 6b) with IC_50_ value 62.86 ± 0.64 µg which is significantly better than BHA (114.58 ± 4.93 µg). However, we found poor scavenging activity in all other plant organs of both *F. praecox* and *M. pruriens*. Among *M. pruriens*, the better scavenging capacity was shown by its leaf (228.79 ± 35.25 µg). Previous studies on other *M. pruriens* species have reported 41% DMPD^⋅+^ scavenging at 40µg^113^ and about 85% DMPD^⋅+^scavenging at 100 µg aqueous extract of its raw seeds^114^. We did not find any previous study on other organs of *M. pruriens* and on any species of *Flemingia* with respect to DMPD^⋅+^ scavenging. In our study, the obtained highest DMPD^⋅+^ scavenging activity of *F. praecox* root indicates that it contain abundant hydrophilic antioxidants as compared to *M. pruriens* which showed significantly lower DMPD^⋅+^ scavenging activity. This indicates that the antioxidant capacity of *M. pruriens* is mostly governed by hydrophobic antioxidants and less by hydrophilic antioxidants.

#### β-carotene bleaching protection assay

Lipid peroxidation protection or peroxyl radical (ROO^⋅^) scavenging property of the plant extracts was assessed by β-carotene bleaching assay. In this assay, the ROO^⋅^ generated by thermal autoxidation of linoleic acid reacts with β-carotene and causes its discolouration. This discolouration is prevented when antioxidants in plants neutralises the ROO^⋅^ to ROOH. This activity is an important indicator of capacity of plant extract to protect fragile sperm membrane susceptible to lipid peroxidation by ROO^⋅^^115^. In our study we observed that at a concentration of 30 µg, *F. praecox* roots have maximum capacity to protect β-carotene from bleaching (29.46 ± 1.40%) followed by *F. praecox* leaf (19.77 ± 2.71%) (Fig. 6c). *M. pruriens* seed protected 14.85 ± 0.30% β-carotene from bleaching. *M. pruriens* stem and root as well as ascorbic acid did not show any protection against the bleaching at this concentration. β-carotene bleaching protection activity of *M. pruriens* was also reported previously where the workers observed 5.4% bleaching protection activity by 100 µg methanolic extract of *M. pruriens*^116^ and 59.35% protection by 200 µg processed extract of another species, *M. gigantia*^117^. No study was found on any *F. praecox* species with respect to their in vitro β-carotene bleaching protection activity. Among the standards, BHA showed the maximum level of β-carotene protection from bleaching (73.01 ± 0.60%) followed by BHT (64.85 ± 0.38%).

#### Ferric ion reducing (Fe^3+^ → Fe^2+^) antioxidant power assay (FRAP)

The reducing capacity of the plant extracts was determined by FRAP assay which is based on the SET mechanism. High reducing capacity of plant extract is an indicator of its potential antioxidant capacity^19^. The results of TAA are presented in Table 4 (SF1b and c). The highest FRAP value was observed in *M. pruriens* seed (A_700_ = 0.194 ± 0.006 absorption units (AU)) followed by *F. praecox* root (A_700_ = 0.143 ± 0.003AU). Among standards, ascorbic acid showed the best reducing capacity (A_700_ = 1.148 ± 0.025AU). In previous studies on *M. pruriens*, FRAP activity was found to be 561 mg ascorbic equivalent/g^68^. However, in our analysis it was observed to have 155.90 ± 5.42 mg ascorbic equivalent/g (SF1a). The FRAP studies on two species of *Flemingia*, *F. vestita* and *F. macrophylla* showed FRAP values, although calculated differently, 9.28 mg GAE/g of hydroalcoholic extract^74^ and an IC_50_ of 23.05 µg/mL of aqueous extract^47^ respectively. Stem and leaf of both *M. pruriens* and *F. praecox* and roots of *M. pruriens* have shown minimum and statistically similar activity indicating their low antioxidant capacity.

**Table 4:** FRAP activity of plant parts of *M. pruriens* and *F. praecox.*

Concentration (µg)	Abs700 ± SD									
	Standard			*M. pruriens*				*F. praecox*		
	Ascorbic acid	BHA	BHT	M. seed	M. leaf	M. stem	M. root	F. root	F. leaf	F. stem
5	0.104 ± 0.011	0.045 ± 0.005	0.037 ± 0.003	0.018 ± 0.003	0.001 ± 0.001	0.011 ± 0.001	0.009 ± 0.002	0.012 ± 0.002	0.015 ± 0.001	0.002 ± 0.000
10	0.201 ± 0.004	0.088 ± 0.005	0.080 ± 0.001	0.029 ± 0.006	0.007 ± 0.001	0.016 ± 0.001	0.013 ± 0.002	0.024 ± 0.002	0.023 ± 0.001	0.007 ± 0.001
20	0.400 ± 0.005	0.155 ± 0.019	0.156 ± 0.001	0.058 ± 0.005	0.016 ± 0.001	0.024 ± 0.001	0.024 ± 0.002	0.042 ± 0.003	0.027 ± 0.002	0.015 ± 0.002
40	0.807 ± 0.013	0.333 ± 0.003	0.315 ± 0.008	0.133 ± 0.010	0.042 ± 0.002	0.049 ± 0.000	0.054 ± 0.002	0.095 ± 0.002	0.054 ± 0.004	0.035 ± 0.001
60	1.148 ± 0.025	0.487 ± 0.004	0.450 ± 0.006	**0.194 ± 0.006**	0.084 ± 0.003	0.074 ± 0.002	0.082 ± 0.002	**0.143 ± 0.003**	0.070 ± 0.002	0.057 ± 0.003
Significance	a	b	c	d	f	fg	f	e	fg	g

Values in bold represent the highest values. Significantly different values are represented with different letters (n = 3; *p* < *0.05*).

#### Total antioxidant activity (TAA)

*To determine* total antioxidant activity (TAA) of the plant extract phosphomolybdenum method was used. This method evaluates both water-soluble and fat-soluble antioxidants from the plant extract^118^. In our study we get the highest TAA value in *F. praecox* root (A_695_ = 0.251 ± 0.002AU) followed by *M. pruriens* seed (A_695_ = 0.137 ± 0.006AU) as can be seen in Table 5 (SF2a and b). However, among the standards used, the highest TAA value was found in ascorbic acid (A_695_ = 0.641 ± 0.005AU) followed by BHA (A_695_ = 0.568 ± 0.03AU) and BHT (A_695_ = 0.317 ± 0.006AU). Previous studies were not found on *M. pruriens* and *F. praecox* in context of the performed assay. This result shows that *F. praecox* root might contain both water-soluble and fat-soluble antioxidants in abundance than *M. pruriens* seeds.

**Table 5:** TAA activity of plant parts of *M. pruriens* and F*. praecox*.

Concentration (µg)	Abs695 ± SD									
	Standard			*M. pruriens*				*F. praecox*		
	Ascorbic acid	BHA	BHT	M. seed	M. leaf	M. stem	M. root	F. root	F. leaf	F. stem
20	0.104 ± 0.007	0.086 ± 0.007	0.067 ± 0.002	0.026 ± 0.001	0.027 ± 0.002	0.015 ± 0.003	0.021 ± 0.005	0.051 ± 0.001	0.018 ± 0.002	0.008 ± 0.005
40	0.228 ± 0.006	0.202 ± 0.010	0.136 ± 0.003	0.055 ± 0.002	0.044 ± 0.002	0.035 ± 0.002	0.037 ± 0.005	0.099 ± 0.004	0.032 ± 0.001	0.016 ± 0.001
60	0.356 ± 0.008	0.278 ± 0.015	0.196 ± 0.006	0.085 ± 0.004	0.066 ± 0.006	0.057 ± 0.001	0.064 ± 0.006	0.148 ± 0.005	0.048 ± 0.001	0.026 ± 0.001
80	0.487 ± 0.010	0.434 ± 0.038	0.255 ± 0.004	0.112 ± 0.008	0.094 ± 0.004	0.073 ± 0.004	0.084 ± 0.001	0.196 ± 0.013	0.066 ± 0.022	0.036 ± 0.003
100	0.641 ± 0.005	0.568 ± 0.030	0.317 ± 0.006	**0.137 ± 0.006**	0.115 ± 0.001	0.092 ± 0.010	0.113 ± 0.005	**0.251 ± 0.002**	0.079 ± 0.011	0.041 ± 0.000
Significance	a	b	c	e	f	g	f	d	g	h

Values in bold represent the highest values. Significantly different values are represented with different letters (n = 3; *p* < *0.05*).

Any plant sample contains hundreds of compounds and its antioxidant property depends upon their physicochemical properties. Therefore, the antioxidant capacity of the plant extract or any sample should not be concluded on the basis of any single antioxidant test model. To evaluate the overall antioxidant potential of the plant extract thus required multiple antioxidant tests to be performed^112^. Our attempt of conducting multiple tests revealed that *F. praecox* root and *M. pruriens* seed have very high antioxidant capacity as revealed by DPPH^⋅^, ABTS^⋅+^, DMPD^⋅+^, FRAP, β-carotene bleaching and TAA assay. This property of *F. praecox* roots and *M. pruriens* seeds might be the major contributing factor in improving male fertility. Moreover, the phytochemically unexplored plant, *F. praecox* which is used by the tribal people have shown exceptional antioxidant properties even better than the conventionally used, *M. pruriens*. In some antioxidant aspects like DPPH, DMPD and β-carotene bleaching assay, it is showing even higher activity than the well-known synthetic antioxidants such as BHA, BHT and ascorbic acid (Table 6) thus giving the promising alternative as a rich source of natural antioxidants to prevent damage caused by oxidative stress to sperms and other important male reproductive physiological parameters.

**Table 6:** The assays performed are either specific in its mechanism to scavenge particular ROS that have a role in male infertility or are used for assessing antioxidants in the samples with different solubility.

Sr. no	Assay	Mechanism	Analogy and function	Role in male fertility	Best results shown by
1	DPPH⋅	HAT and SET, assesses hydrophobic antioxidants	Free radicals, reducing ability	Involved in idiopathic infertility^119^	BHA = AA > *F. praecox* root > BHT > *M. pruriens* seed
2	ABTS⋅ +	HAT and SET, assesses hydrophilic and hydrophobic antioxidants	Free radicals, reducing ability	Involved in idiopathic infertility^119^	BHA > AA = BHT > *F. praecox* root > *M. pruriens* seed
3	DMPD⋅ +	HAT and SET, assesses hydrophilic antioxidants	Free radicals, reducing ability	Involved in idiopathic infertility^119^	AA > *F. praecox* root > BHA > *M. pruriens* leaf
4	β-carotene bleaching protection	Assesses lipid peroxidation preventing antioxidants	ROO⋅, membrane protection	Damages sperms by lipid peroxidation of membranes^120^	BHA > BHT > *F. praecox* root > *F. praecox* leaf > *M. pruriens* seed
5	FRAP	SET, assesses metallic free radical scavenging capacity	Iron chelation and reducing power that can work against H_2_O_2_, ^1^O_2_, HO^⋅^, and O^⋅−^	Involved in sperm DNA damaging HO^•^ generation, idiopathic infertility^121^	AA > BHA > BHT > *M. pruriens* seed > *F. praecox* root
6	TAA	HAT and SET, assesses total antioxidants from broad spectrum of samples	Metal chelation and Reducing power that can work against H_2_O_2_, ^1^O_2_, HO^⋅^, and O^⋅−^	Involved in idiopathic infertility^119,121^	AA > BHA > BHT > *F. praecox* root > *M. pruriens* seed

Overall, our results shows that, except FRAP assay, in all the assays *F. praecox* roots have better antioxidant capacity than *M. pruriens* seeds. *AA* ascorbic acid.

### DNA damage protection activity

**Figure 7:**
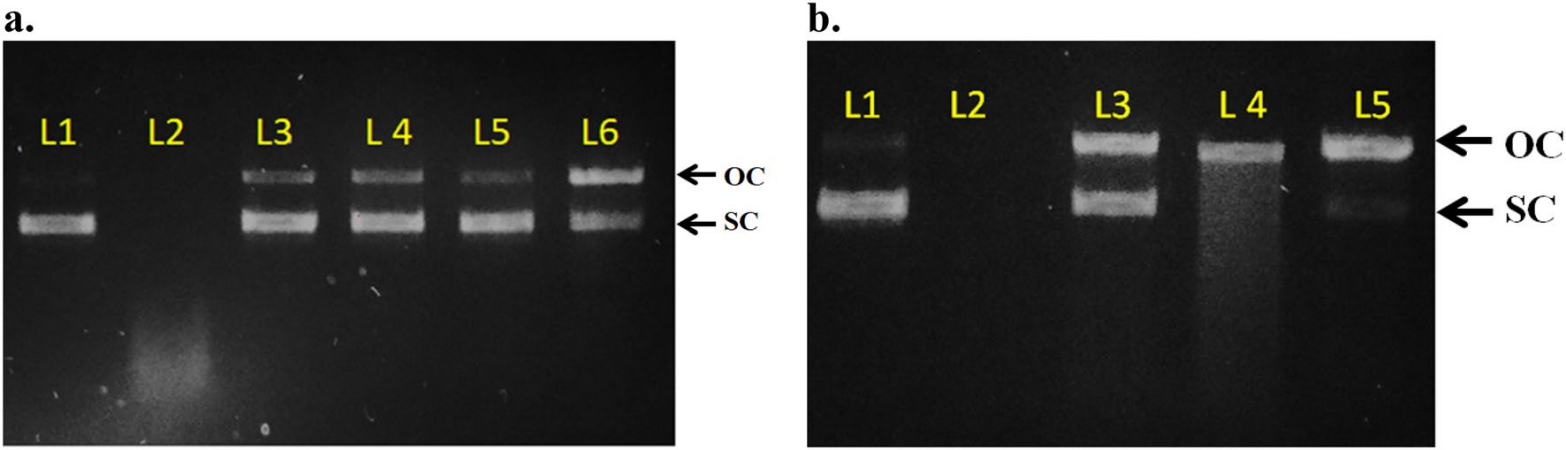
DNA damage protection activity of methanolic extracts of *M. pruriens* seeds (**a**) and *F. praecox* roots (**b**). L1 = Plasmid DNA (pDNA), control; L2 = pDNA + Fenton’s Reagent. In (**a**) L3 = pDNA + FR + *M. pruriens* Seed Extract; L4 = pDNA + FR + *M. pruriens* Leaf Extract; L5 = pDNA + FR + *M. pruriens* Stem Extract; L6 = pDNA + FR + *M. pruriens* Root Extract. In (**b**) L3 = pDNA + FR + *F. praecox* Root Extract; L4 = pDNA + FR + *F. praecox* Leaf Extract; L5 = pDNA + FR + *F. praecox* Stem Extract. Arrows indicate distinct forms of plasmid DNA: OC (open circular); SC (supercoiled).

**Figure 8:**
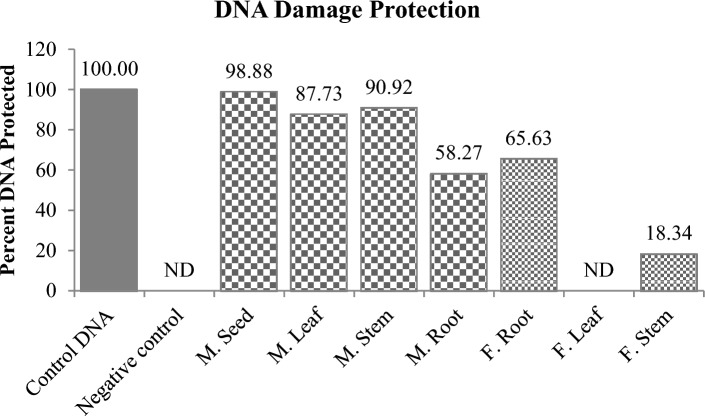
DNA damage protection activity of methanolic extracts of *M. pruriens* and *F. praecox* plant organs (*ND* not detected).

DNA protection capacity of all the plant extracts against damaging agent, Fenton’s reagent was assessed by agarose gel electrophoresis. Fenton’s reagent generates highly reactive, DNA damaging hydroxyl radical (HO^**⋅**^). This radical is known to damage the DNA by oxidizing 2-deoxyribose to malonaldehyde^116^. Therefore, the plant antioxidants are used to assess their HO^**⋅**^ radical scavenging capacity and to protect DNA against the damage caused by the radical (Fig. 7a and b). The highest DNA protection was governed by *M. pruriens* seed extract which protected 98.88% DNA at 50 µg concentration followed by its stem and leaf which protected 90.92% and 87.73% respectively. In *F. praecox*, its roots protected the maximum 65.63% DNA followed by its leaf which showed 18.34% protection (Fig. 8 and SF3). The *F. praecox* stem did not show any protection against DNA damage. Previous studies shows that *M. pruriens* have capacity to scavenge HO^**•**^ radical and protect DNA in dose dependent manner^116^. One study shows the methanolic extract of the *M. pruriens* has DNA protection capacity at IC_50_ value of 38 µg^66^. Not much work has been done before on DNA protection activity of *F. praecox*. However, in one study where Kim et al. isolated bioactive compound, auriculasin form *F. philippinensis* which showed 90.9% DNA protection at 60 µM concentration^83^.

### Correlation study

**Figure 9:**
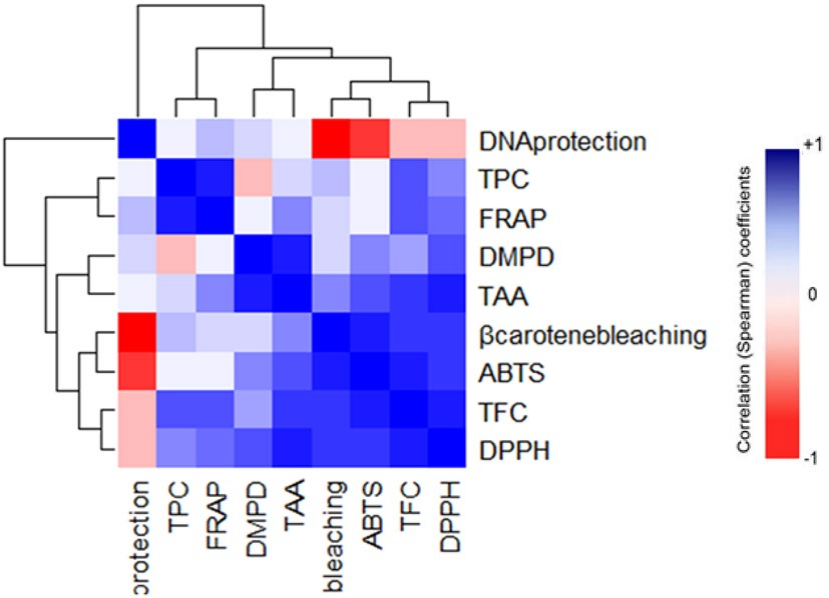
Similarity matrices (correlation study) between the antioxidant assays that were represented as heatmap and hierarchical clustering tree.

The different antioxidant assays studied represent their different modes of action towards different ROS and FR^84^. To know whether their modes of actions are correlated to their properties of eliminating ROS and FR, we studied the correlation among their activity by using Pearson’s correlation (Fig. 9 and SF4). The TPC was found positively correlated to only TFC (r = 0.76) at *p* < *0.05* significance level possibly because phenolics and flavonoids are structurally related. On the other hand, flavonoids have shown strong positive correlation (*p* < *0.01*) with DPPH (r = 0.92) and ABTS (r = 0.90) showing their ability of HAT and SET to eliminate ROS and FR. Previously, researchers have also found good correlation between total polyphenols (including flavonoids) and DPPH, ABTS activities but have noted comparatively lower correlation with DMPD radical scavenging activity^122^. Moreover, flavonoids also displayed good correlation with β-carotene bleaching, TAA and FRAP at *p* < *0.05* significance level representing that they are involved in lipid peroxidation protection. These results indicate that flavonoids are better antioxidants with a wider spectrum of scavenging mechanisms than phenols which is also evident from the result of previous work^21^. TAA is also strongly correlated with DPPH (r = 0.89) and DMPD (r = 0.88) at *p* < *0.01* significance level. This might be attributed to capacity of TAA assay to measure both hydrophobic and hydrophilic antioxidants^118^ and thus showing cumulative activity based on the principle of both DMPD and DPPH assay which are known to be more specific to hydrophilic and hydrophobic antioxidants respectively^19^. Similarly, β-carotene bleaching and ABTS activity are strongly correlated (r = 0.90). This indicating the similar mechanism of HAT might be required for scavenging ROO^⋅^ in β-carotene bleaching assay and ABTS assay^19^. Finally, DNA damage protection although is positively correlated with all the assays except β-carotene bleaching and ABTS, its correlation was found to be non-significant. This suggesting that, DNA damage protection assay which is mainly based on HO^•^ scavenging property of the compound^123^ may be having quite different mechanism of action towards scavenging HO^⋅^ than other assays tested. FRAP assay is based on ferric ion reducing capacity of antioxidants^19^ and under OS ferric ions can generate DNA damaging HO^⋅^ by Fenton’s reaction^116^_._ Our results of FRAP assay showed best activity in *M. pruriens* seeds and similar results were also observed in DNA damage protection assay indicating both the activities are correlated and *M. pruriens* seed’s metabolites may have active involvement in improving male fertility by protecting sperm DNA from HO^⋅^.

Collectively, our results indicate that flavonoids are the major contributing factor for the antioxidant capacity of the plant extract. Therefore, *F. praecox* roots being the most flavonoid rich part of the plant can serve as a better source of antioxidants than conventionally used *M. pruriens* seeds to protect from ROS and FR and to repair and improve the male fertility.

### PDE5 and arginase inhibition activity

**Figure 10:**
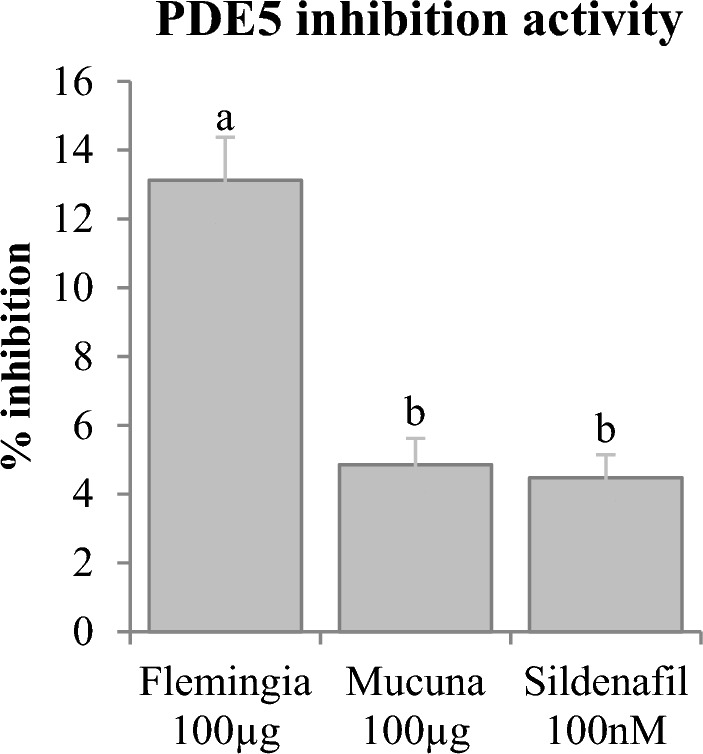
PDE5 inhibition activity of methanolic extract of *F. praecox* and *M. pruriens*. Different letters represent significant differences at the *p* < *0.05* level.

Both PDE5 and arginase enzymes are considered as negative regulators of erection and their over activity or expression can cause erectile dysfunction by independent mechanisms. PDE5 is known to terminate cyclic nucleotide signalling required to mediate relaxation of smooth muscle necessary for the penile erection^124^. Medicines like sildenafil, vardenafil and tadalafil are effective inhibitors of PDE5 thus helping in the management of erectile dysfunction^125^. Here we have attempted to study whether our plant extracts have any capacity to inhibit the PDE5 activity (Fig. 10). Our study revealed that *F. praecox* root extract at 100 µg concentration inhibited the PDE5 activity by 13.12% whereas at the same concentration *M. pruriens* showed inhibition activity of 4.85%. This result suggests that *F. praecox* might contain more effective PDE5 inhibitors than *M. pruriens*. However, sildenafil citrate has shown nearly similar inhibition percent to *M. pruriens* at 100 nM concentration (4.48%).

Another biomarker for erectile dysfunction studied is arginase which works by competing for the l-arginine, the substrate for the nitric oxide synthase (NOS) needed for the synthesis of nitric oxide (NO). NO is an important molecule for penile cavernosal tissue relaxation and erection^126^. Therefore, arginase inhibitors can enhance L-arginine bioavailability to NOS. Our study showed that both *M. pruriens* seed and *F. praecox* root extract have nearly similar arginase inhibition capacity with their IC_50_ value calculated to be 144.41 ± 46 µg and 146.20 ± 29.68 µg respectively (Table 7).

**Table 7:** Statistically similar values of arginase inhibition activity (at *p* < *0.05* level) of methanolic extract of *F. praecox* and *M. pruriens*.

	*F. praecox*	*M. pruriens*
Arginase inhibition activity (IC_50_)	146.20 ± 29.68 µg	144.41 ± 46 µg

## Conclusion

As it is evident from the previous work that ROS has a huge impact on the male fertility and its effect can be reversed with the help of antioxidants from natural sources like *M. pruriens*. Therefore, the aim of the present investigation was set to examine a similar role of a less explored but traditionally effective and endemic plant, *F. praecox* and its activity was compared with the activity of *M. pruriens*. This aim was investigated with the help of examining their antioxidant parameters like phenolic, flavonoid content, DPPH, ABTS, DMPD radical scavenging capacity, β-carotene bleaching protection, FRAP and TAA activity along with DNA damage protection capacity. The second aim was to directly examine comparative inhibition potential of both the plants against infertility markers PDE5 and arginase enzymes.

The study has identified *F. praecox* is having better antioxidant activity than *M. pruriens* in majority of the antioxidant assays suggesting that antioxidant potential of *F. praecox* may be the contributing factor for its fertility improving activity. Another significant observation about *F. praecox* is that its radical scavenging capacity is better than artificial antioxidants thus implicating its further use as a source of dietary antioxidants or can be used in combination with available antioxidants for better synergistic effects for improving the male fertility. The presence of a higher number of phenolic compounds in *F. praecox* roots compared to *M. pruriens seeds*, along with the diverse mechanisms by which these compounds positively influence male fertility, emphasises their potential role in enhancing male reproductive health. Finally. the finding of better PDE5 inhibition activity and similar arginase inhibition values of *F. praecox* in relation to its counterpart, *M. pruriens* is again encouraging for its further preclinical and clinical trials to study its actual potential.

## Materials and methods

### Plant material collection and processing

For collection of plants, all relevant permits or permissions have been obtained. The study also complies with local and national regulations. *F. praecox* C.B. Clarke Ex Prain was collected from Gadchiroli district of Maharashtra, India and identified by D. L. Shirodkar, botanist from Botanical Survey of India (BSI), Pune and deposited in the herbarium of BSI, Pune with identification No. BSI/WRC/Iden. Cer./2021/0911210004872. *F. praecox* is extremely rare in the natural habitat therefore, very few seeds of it were collected from Gadchiroli district of Maharashtra, India and then it was planted and grown for two years till its further successful seed setting has occurred. Later, its leaf, stem and roots were harvested, cleaned, washed, chopped, dried in a hot air oven at 45 °C and powdered in a mechanical grinder. *M. pruriens* L. was collected from RTM Nagpur University Educational Campus, Nagpur, India and identified by Prof. N. M. Dongarwar, taxonomist in Department of Botany, RTM Nagpur University, Nagpur (identification No. 187). Its seeds, leaves, stem and roots were collected and processed in a similar way like that of *F. praecox*. All samples were extracted in methanol by soxhlet. Also the seed of *M. pruriens* and roots of *F. praecox* were extracted sequentially in different solvents like n-hexane, ethyl acetate, chloroform, acetone and methanol with their increasing polarity then filtered and used for further analysis.

### Preliminary phytochemical analysis

Preliminary phytochemical tests were done as per the standardized protocols^127–129^.

#### Test for phenols

##### Ferric chloride test

Three to four drops of 5% FeCl_3_ solution was added in 2 mL of crude extract of plants. Appearance of bluish black colour confirms the presence of phenols.

#### Test for flavonoids

##### Lead acetate test

1 mL of 10% lead acetate solution was added to 1–2 mL of plant extract. The appearance of blue colour confirms the presence of flavonoids.

##### Shinoda test

For this test, in the aqueous extract of plants some pieces of magnesium metal ribbons were added followed by addition few drops of concentrated HCl which within a minute or two gives pink, crimson or magenta colour that shows presence of flavonoids.

##### Alkaline reagent test

For this test 2 mL of 2% NaOH was added to 1–2 mL of aqueous extract of plants that give intense yellow colour. Addition of 3 mL of 5% HCl to it turns reaction mixture colourless indicates presence of flavonoids.

#### Test for alkaloids

##### Hager’s test

Freshly prepared Hager’s reagent (1 g picric acid in 100 mL hot water) when added to plant extract gives yellow precipitate indicating presence of alkaloids.

##### Dragendorff’s test

Few drops of Dragendorff’s reagent were added to the plant extract which gives orange, red or creamy precipitate confirms presence of alkaloids.

##### Mayer’s test

Mayer’s reagent (potassium mercuric iodide) when reacted with alkaloids in plant extract (2 mL) gives yellow, whitish or creamy precipitate.

##### Wagner’s test

1 mL of Wagner’s reagent added to 2 mL of plant extract, reddish brown precipitate confirms the presence of alkaloids.

#### Test for steroids

##### Salkowski test

In this test, 2 mL of extract is used and 2 mL chloroform and 1–2 mL concentrated sulphuric acid were added to it, the reddish brown colour at the junction of aqueous and chloroform layer indicates presence of steroids.

#### Test for tannins

##### Bramer’s test

2-3drops of 5% FeCl_3_ solution was added to diluted plant extract. Appearance of green or bluish black precipitate indicates presence of tannins.

##### Lead acetate test

In this test, to the 2 mL of extract 10% lead acetate solution was added. Appearance of white precipitation confirms the presence of tannins.

##### Potassium dichromate test

In 2 mL of plant extract, formation of red or dark coloured precipitate after addition of 10% potassium dichromate confirms the presence of tannins.

##### Gelatin test

1 mL of 1% gelatin solution in 10% NaCl was prepared and added to 2 mL of extract. Formation of white precipitate indicates presence of tannins.

#### Test for saponins

##### Foam test

5 mL of aqueous extract or 500 mg of dry extract was heated and shaken with 5 mL distilled water. Foam produced persisted for 10 min indicates presence of saponins.

##### Olive oil test

In 5 mL of extract a few drops of olive oil was added and the solution was shaken vigorously. Formation of emulsion confirms presence of saponins.

#### Test for glycosides

##### Keller-kiliani test

To the 2 mL of plant extract 1 mL of glacial acetic acid was added followed by addition of a few drops of FeCl_3_ and at the end 1 mL of H_2_SO_4_ added slowly and the solution allowed to settle. A reddish brown colour ring appears at the junction of two layers and the upper layer turns bluish green. These results suggest the presence of cardiac steroidal glycosides (aglycon).

##### Legal’s test

2 mL of concentrated extract mixed with 2 mL of pyridine, few drops of 2% freshly prepared sodium nitroprusside solution and few drops of 20% NaOH. Blue or pink coloration indicates presence of aglycon moiety.

##### Liebermann’s test

2 mL of extract was heated with 2 mL of acetic anhydride. After its cooling a few drops of concentrated H_2_SO_4_ was added from the sides of the test tube. Appearance of the blue or green colour precipitate indicates presence of glycosides.

#### Test for terpenoids

##### Acetic anhydride test

2 mL of acetic anhydride was added to 2 mL of extract followed by addition of 2–3 drops of concentrated H_2_SO_4._ The deep red coloration indicated the presence of terpenoids.

##### Chloroform test

In this test, to the 2 mL of plant extract, 2 mL chloroform was added and the solution was evaporated in a water bath to make its concentrate. Later 3 mL H_2_SO_4_ was added and the solution was boiled. The grey colour will appear when the terpenoids are present.

#### Total phenol content (TPC)

TPC was estimated by Folin-ciocalteu method^130^. In brief, 2.5 mL of 10% Folin-ciocalteu reagent and 2 mL of 7.5% sodium carbonate were added to 500 µg of extract. The reaction mixture was incubated at 45 °C for 45 min and the blue coloured phosphomolybdic/phosphotungstic acid complex was measured at 760 nm. The TPC value was calculated using gallic acid standard and presented as mg GAE/g of extract.

#### Total flavonoid content (TFC)

TFC was determined by aluminium chloride method^131^ with slight modification. 200µL of 5% sodium nitrite was added to 200 µg of extract and allowed to react for 5 min. 300µL of 10% aluminium chloride was added to the mixture and after 5 min, 2 mL of 1 M NaOH was added and the absorbance of the orange-red aluminium complex was taken at 510 nm. The TFC value was calculated using the quercetin standard and presented as mg QE/g of extract.

### Phenol and flavonoid detection in plant fractions by HPLC–MS/MS analysis

One gram of dried *Flemingia* root powder and *Mucuna* seed powder were macerated in HPLC grade Methanol for 48 h. The extract was filtered by Whatman filter paper no. 1 and clear filtrate was used for the metabolome analysis by HRLC-MS/MS. The metabolomics data generated was then searched for the phenol and flavonoid compounds. Detailed set up procedure for HPLC–MS/MS instrument for the analysis is given in supplementary data file.

### 2, 2-Diphenyl-1-picrylhydrazyl radical (DPPH^⋅^) scavenging assay

DPPH⋅ scavenging assay was done as per the procedure explained by Tuba and Gulcin^132^ with some modification as per Kedare and Singh^133^. Purple coloured DPPH^**⋅**^ solution was prepared in methanol till the absorbance was achieved to 0.950 ± 0.025 at 517 nm. 3 mL methanol was added to 4, 8, 12, 16 and 20 µg of plant extract followed by addition of 1 mL DPPH^**⋅**^ solution. The reaction mixture vortexed and incubated at RT for 30 min in the dark. Absorbance of the pale yellow hydrazine product measured at 517 nm with blank containing only methanol. IC_50_ values of samples were calculated along with the ascorbic acid, BHA and BHT standards.

### 2, 2-azinobis (3-ethylbenzothiazoline-6-sulfonic acid radical (ABTS^⋅+^) scavenging assay

ABTS^⋅+^ scavenging activity of the plant extracts were determined by first generating ABTS radical cation (ABTS^⋅+^) by mixing 7 mM ABTS and 2.45 mM potassium persulfate in deionized water and kept at room temperature for overnight (12–16 h) and finally the absorbance of ABTS^⋅+^ was adjusted to 0.750 ± 0.025 at 734 nm. Later 3 mL methanol and 1 mL ABTS^⋅+^ solution was added to 2, 4, 6, 8 and 10 µg of plant extract. After 10 min of incubation at RT, the absorbance of decolorized/scavenged ABTS^⋅+^ was measured at 734 nm with blank containing only methanol^134^. IC_50_ values of samples were calculated along with the ascorbic acid, BHA and BHT standards.

### N, N-dimethyl-p-phenylenediamine dihydrochloride radicle (DMPD^⋅+^) scavenging assay

DMPD cation radical (DMPD^⋅**+**^) generated by reacting DMPD with ferric chloride in acetate buffer. For this 500µL of 100 mM DMPD was added to 50 mL of 0.1 M acetate buffer (pH 5.3) and then 100µL of ferric chloride added to generate DMPD^⋅**+**^. Finally the absorbance of this solution was adjusted by using acetate buffer or ferric chloride to 0.900 ± 0.100 at 505 nm. Now, 2 mL of the DMPD^**⋅+**^ solution was added to 10, 20, 30, 40 and 50µL of extract and incubated at RT for 10 min and discoloration is noted at 505 nm by using acetate buffer as blank^135^. IC_50_ values of samples were calculated along with the ascorbic acid, BHA and BHT standards.

### Ferric ion reducing (Fe^3+^ → Fe^2+^) antioxidant power assay (FRAP)

The FRAP assay for formation of intense perl’s prussian blue complex of the Fe^2+^–ferricyanide complexes from yellow coloured Fe^3+^–ferricyanide complexes by the reducing power of plant extract was also performed^132^. Briefly, different concentrations of plant extracts (5, 10, 20, 30, 40 and 60 µg) was taken and reacted with 2.5 mL of 1% potassium ferricyanide in 2.5 mL sodium phosphate buffer (0.2 M; pH 6.6) and incubated at 50 °C for 20 min. Then 2.5 mL of 10% trichloroacetic acid was added. 2.5 mL of this reaction mixture was taken then diluted with 2.5 mL distilled water and 0.5 mL of 0.1% ferric chloride was added. The absorption of the complex was measured at 700 nm.

### β-carotene bleaching protection assay

A β-carotene bleaching assay was done by using protocol of Duan et al.^136^. Shortly, 1 mg/mL β-carotene solution was prepared in chloroform and 4 mL of it was added to 45µL of linoleic acid and 365 µL of tween-20. Chloroform was evaporated and slowly 100 mL oxygenated distilled water was added and vortexed to form emulsion and to initiate the β-carotene bleaching. 4 mL of it was added to 30 µg of the plant extract and delay in discolouration by plant extract was noted after 60 min of incubation for 45–50 °C at 470 nm.

### Phosphomolybdenum method for total antioxidant activity (TAA)

In this method different concentration of plant extract (20, 40, 60, 80 and 100 µg) was reacted with 5.4 mL phosphomolybdenum reagent made up of 28 mM sodium phosphate, 4 mM ammonium molybdate and 0.6 M sulfuric acid. The reaction mixture then incubated at high temperature of 95 °C for 90 min, cooled at room temperature and subsequently the absorbance of green phosphate/Mo(V) complex formed noted at 695nm^118^.

### DNA damage protection activity

DNA damage protection capacity of the plant extract from Fenton’s reagent was determined by using plasmid DNA as explained by Kim^83^ with some modifications. Briefly, in the sequence, reaction mixture of 3 µl of Plasmid DNA (0.35 µg/mL), 9 µl of 50 mM sodium phosphate buffer (pH 7.4), 2 µl of 1 mM FeSO_4_, 50 µg of sample and 3 µl of 30% H_2_O_2_ was prepared. Then the reaction mixture incubated at 37 °C for 30 min in the dark. 5 µl of it was loaded in 0.8% agarose gel with 1 µl of 6 × DNA loading buffer for electrophoresis for 60 min at 85 V and 90 mA. The bands generated were analyzed by using Image Lab software and percent DNA protection was calculated by comparing with control containing only plasmid DNA.

### In vitro PDE5 inhibition activity

Rat lung homogenate was used as a source of PDE5 enzyme^137^. The homogenate (10% w/v) was centrifuged at 13,000 rpm for 20 min and supernatant was used as a source of enzyme for inhibition assay. The reaction mixture was prepared in the following sequence. 100 µg of plant extract in 5% DMSO was added to 2 mL of 20 mM Tris–HCl (pH 8.0) containing 5 mM MgCl_2_ followed by the addition of 100µL of enzyme extract. Finally, 100µL of 5 mM 4-nitrophenyl phenylphosphonate substrate was added to initiate the reaction. After incubation of 60 min at 37 °C, the absorbance of the hydrolysed product from substrate was noted at 400nm^138^ using 5% DMSO as blank and compared with the sildenafil as a positive control.

### In vitro arginase inhibition activity

Arginase inhibition capacity of the plant extract was determined by using lung tissue homogenate as a source of arginase by method developed by Iyamu et al.^139^. The reaction mixture including 100µL enzyme extract, 100µL of 100 mM MnCl_2_, 1 mL of 50 mM Tris–HCl (pH 7.5) and 50µL of 0.5 M arginine substrate/ substrate with 50µL plant extract (1 mg/mL)/ substrate with 50µL DMSO (5%) incubated at 37ºC for 60 min. Then the reaction was stopped by adding 1 mL of 0.72 M HCl, the solution was centrifuged for 5 min at 5000 rpm. 1 mL of supernatant was mixed with 2 mL of 6% ninhydrin in ethanol. Finally, the solution was incubated at 60 °C for 30 min, cooled at RT and the formation of a reddish complex was noted at 505 nm. The inhibition percent was calculated by comparing the result of sample with control.

All experimental protocols were approved by the Institutional Animal Ethical Committee of Smt. Kishoritai Bhoyar College of Pharmacy, Kamptee, Nagpur, Maharashtra (IAEC approval No. 853/IAEC/22-23/23). Quarantine procedures and animal maintenance followed the recommendations of CPCSEA (Committee for the Purpose of Control and Supervision of Experiments on Animals) guidelines for laboratory animal facilities, and the methods are reported in accordance with ARRIVE guidelines.

### Statistical analysis

All the analyses were performed in triplicate experiments (n = 3). The results of TPC, TFC, TAA and FRAP were calculated as mean of observations ± SD. Whereas for DPPH, ABTS and DMPD radical scavenging activities, the means of IC_50_ ± SD was calculated. β-carotene bleaching assay, DNA damage protection assay and in-vitro PDE5 and arginase inhibition capacity were calculated as mean of percent protection/inhibition ± SD. For defining the statistical significance between the observations, analysis of variance (ANOVA) and Tukey’s post-hoc test was applied (*p* < *0.05*) and for Pearson’s correlational studies between antioxidant tests, *multcompview* and *metan package* in R were used.

### Supplementary Information

Supplementary Information.

## Data Availability

All data generated or analysed during this study are included in this published article and its supplementary information file.
