# Supplementary material for: Antioxidant activity of Flemingia praecox and Mucuna pruriens and their implications for male fertility improvement
Source: Sci Rep. 2023 Nov 8;13:19360. doi: 10.1038/s41598-023-46705-9 (PMC10632466; doi:10.1038/s41598-023-46705-9)
Supplement: Supplementary file 1 — Supplementary Information. [file 41598_2023_46705_MOESM1_ESM.pdf]

Antioxidant activity of *Flemingia praecox* and *Mucuna pruriens* and their implications for male fertility improvement

Shravan D. Kumbhare[1†], Sanghadeep S. Ukey[1,3] and Dayanand P. Gogle[1,2*]

[1]Post Graduate Teaching Department of Botany, RTM Nagpur University, Nagpur-440033, Maharashtra, India; [2]Post Graduate Teaching Department of Molecular Biology and Genetic Engineering, RTM Nagpur University, Nagpur-440033, Maharashtra, India; [3]Department of Botany, Lokmanya Tilak College, Yawavatmal-445304, Maharashtra, India.

[*]dr.dayanand.gogle@nagpuruniversity.nic.in

Supplementary Data

Supplementary Figure 1a (SF1a). FRAP assay of *F. praecox* and *M. pruriens* (Ascorbic acid equivalent)

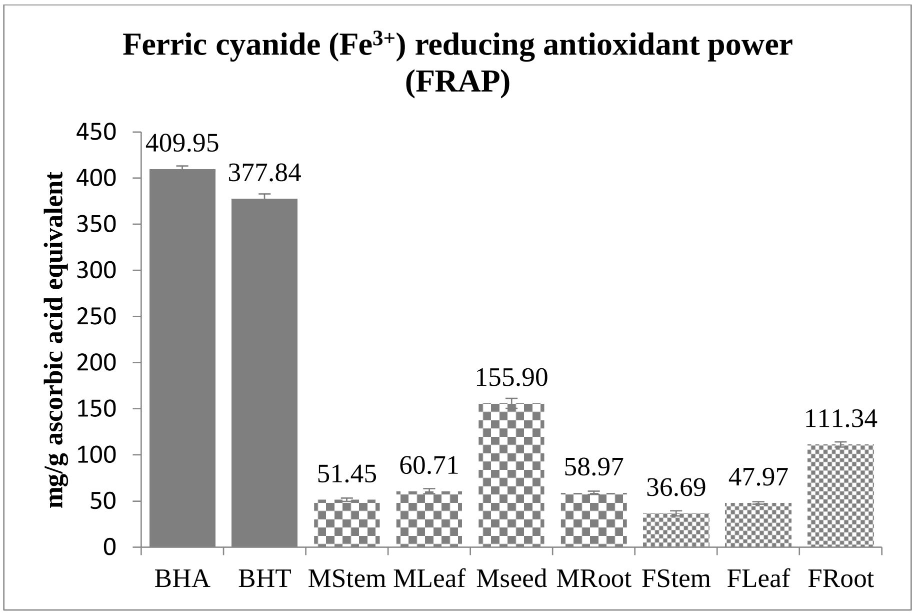

SF1b. FRAP of *M. pruriens*

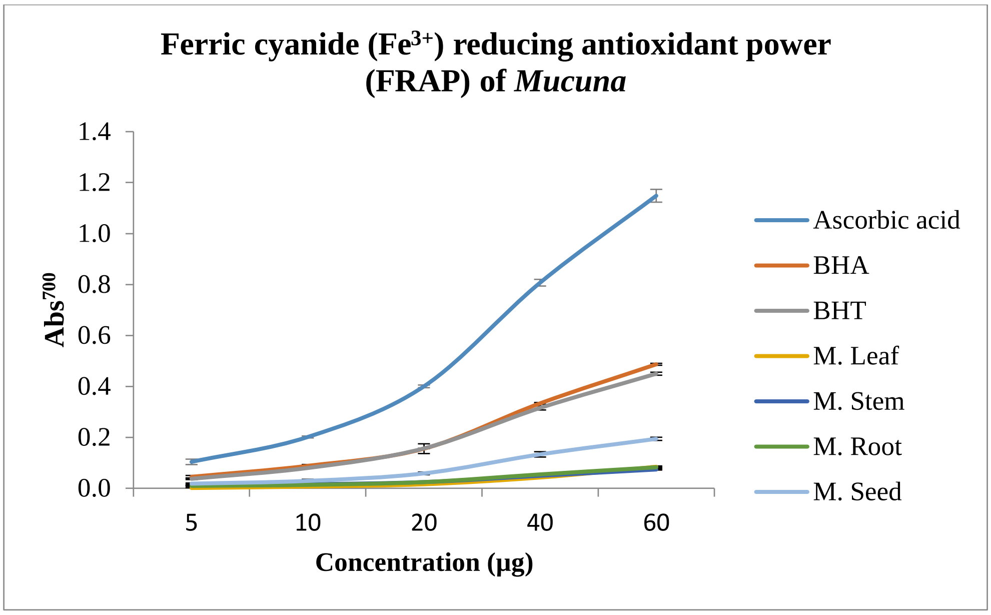

SF1c. FRAP of *F. praecox*

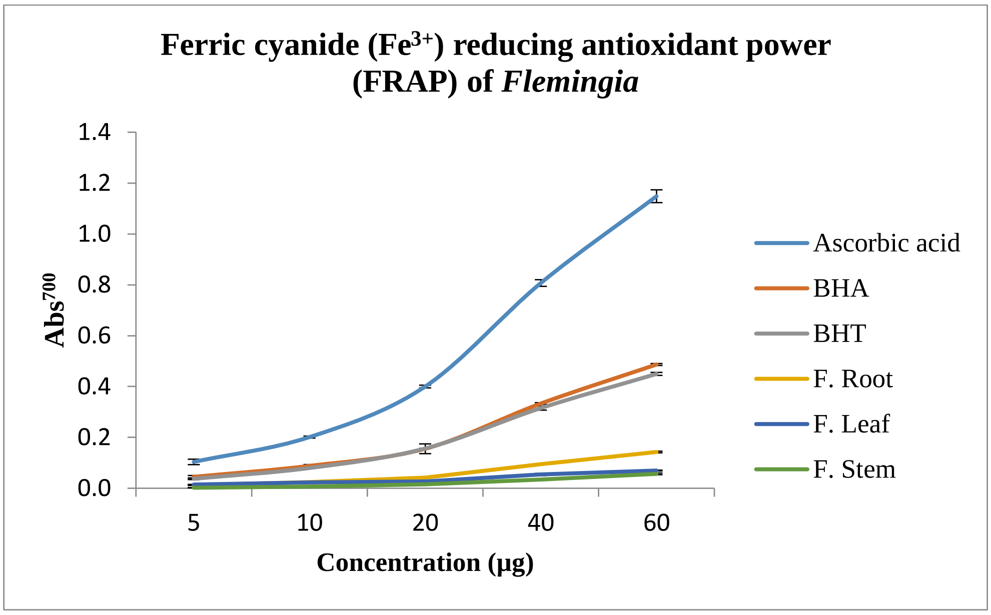

SF2a. TAA of *M. pruriens*

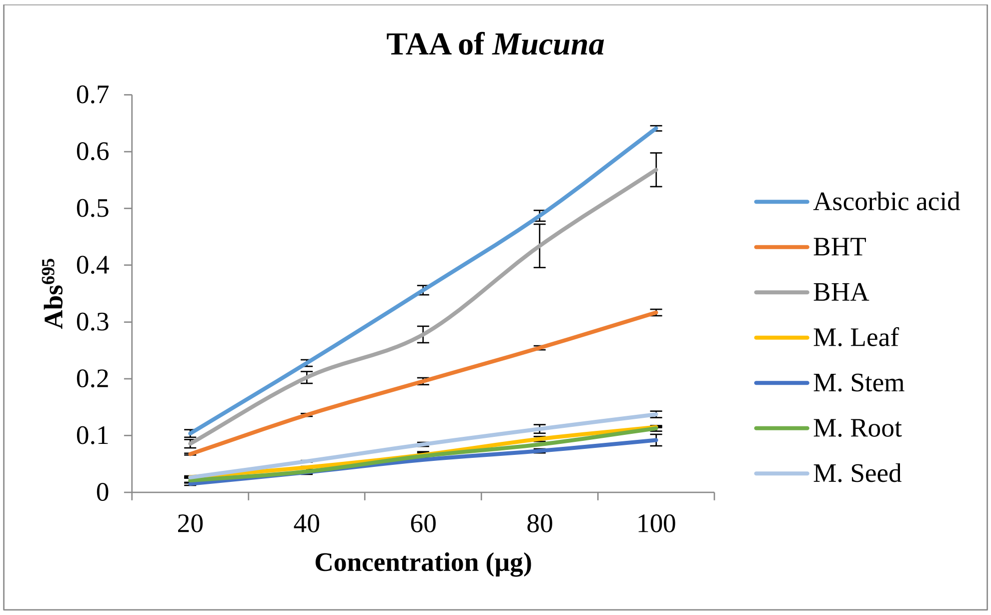

SF2b. TAA of *F. praecox*

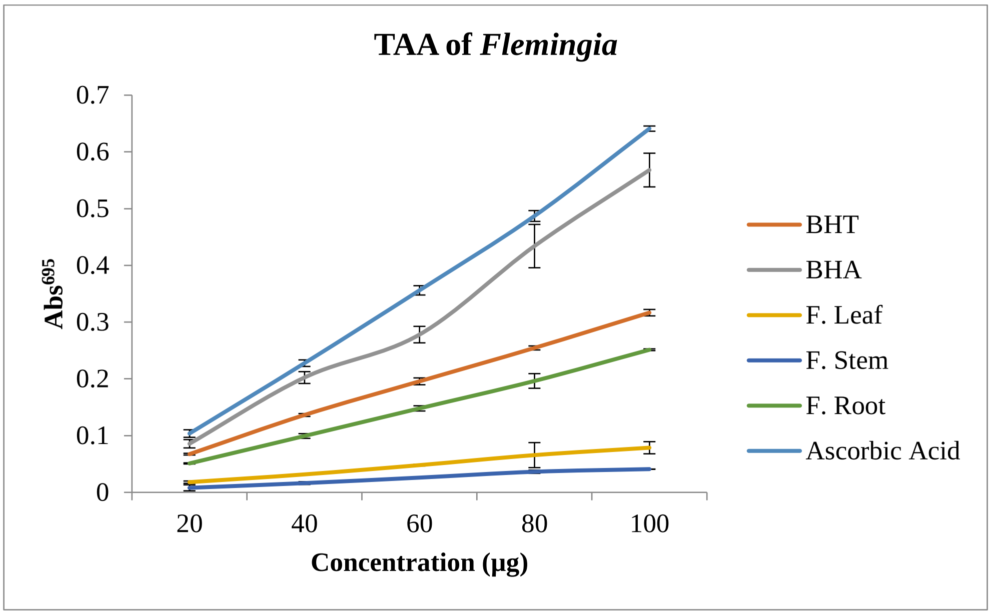

SF3. Densitometric analysis of gel by Image lab software for DNA damage protection study of *F. praecox* and *M. pruriens*

F. praecox

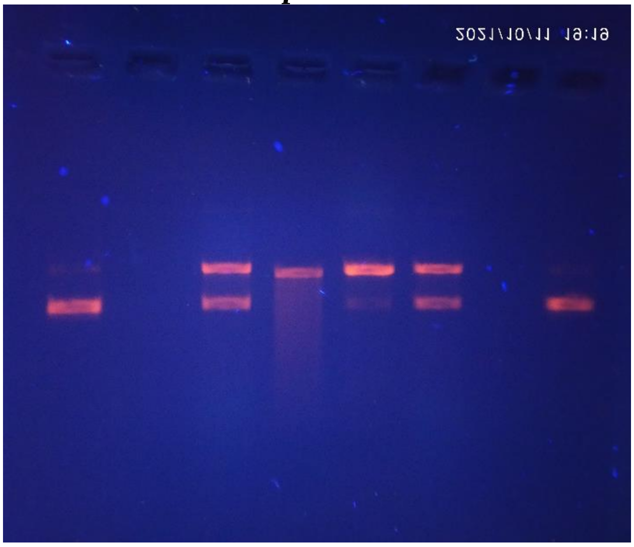

Lane 1

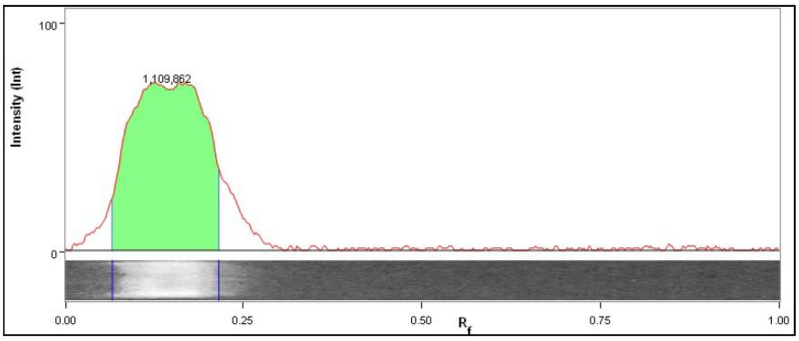

L1= Plasmid DNA (pDNA), control

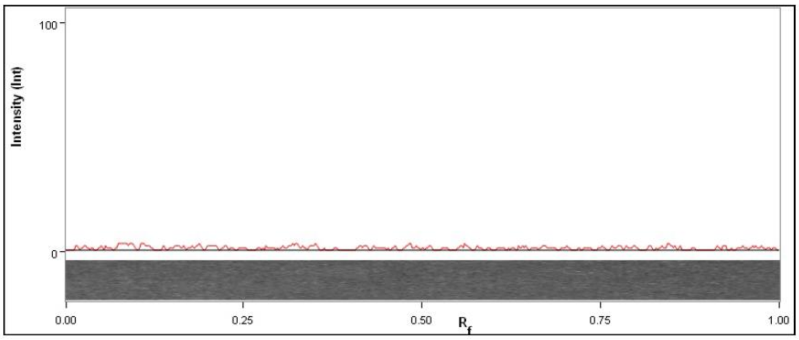

L2= pDNA+Fenton's Reagent

Lane 3

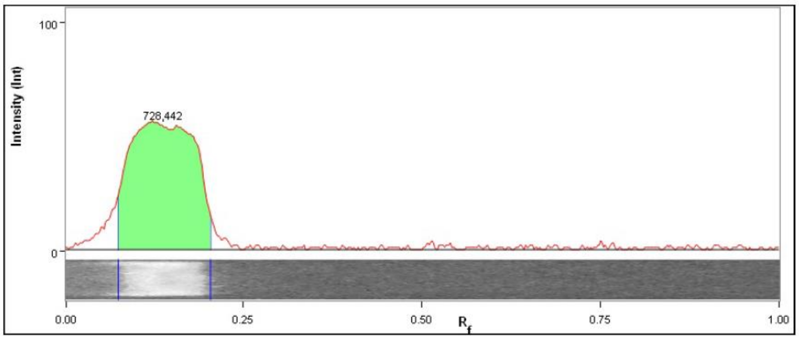

L3= pDNA + FR + *Flemingia* Root Extract

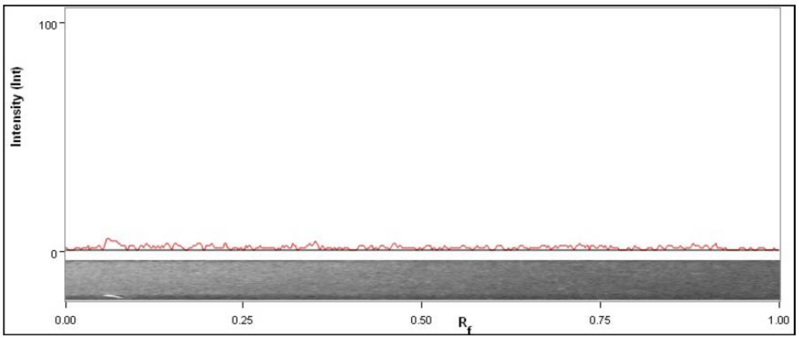

L4= pDNA + FR + *Flemingia* Leaf Extract

Lane 5

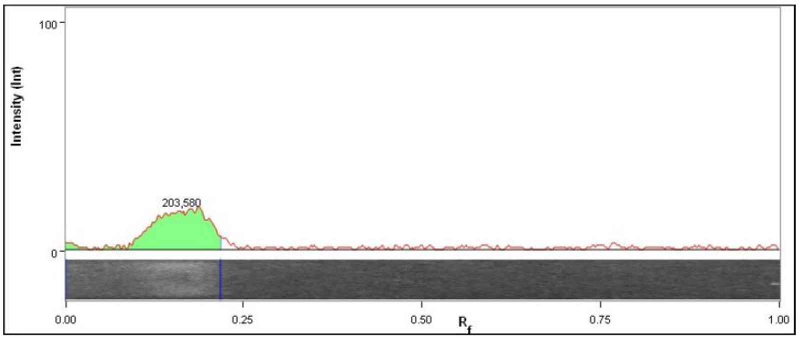

L5= pDNA + FR + *Flemingia* Stem Extract

M. pruriens

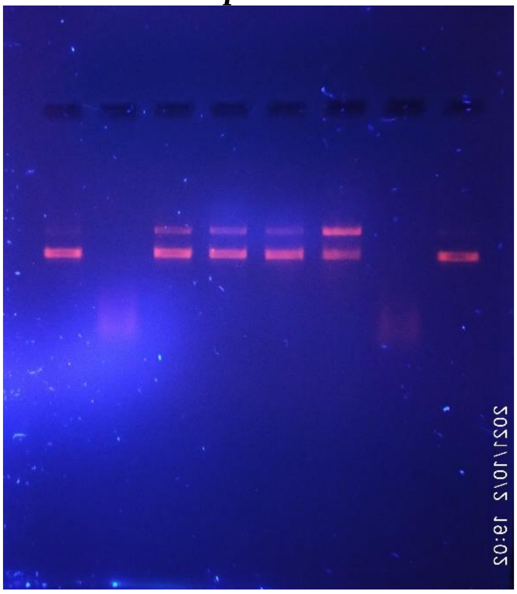

Lane 1

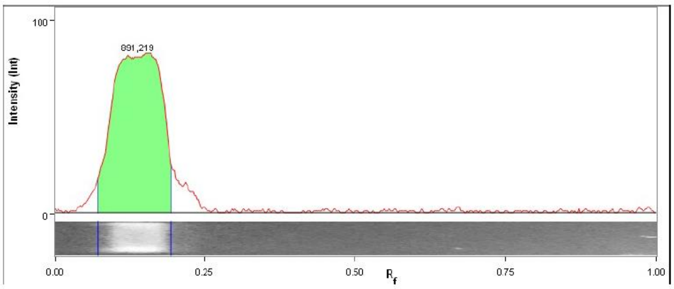

L1= Plasmid DNA (pDNA), control

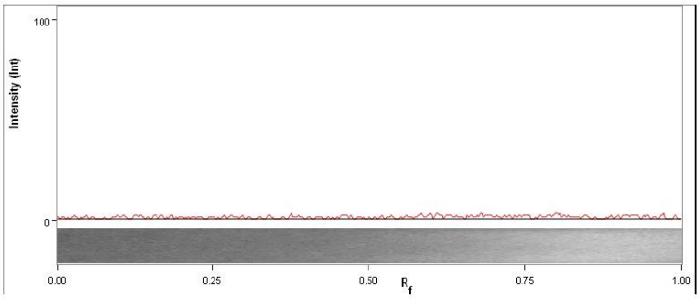

L2= pDNA+Fenton's Reagent

Lane 3

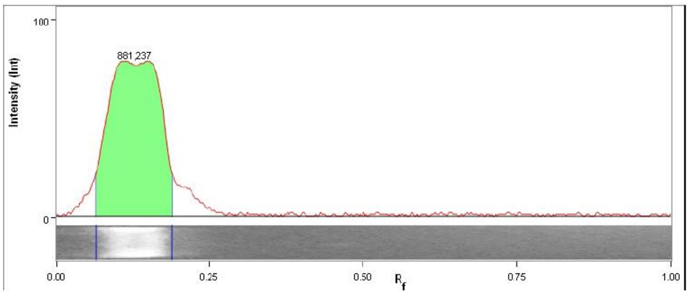

L3= pDNA + FR + *Mucuna* Seed Extract

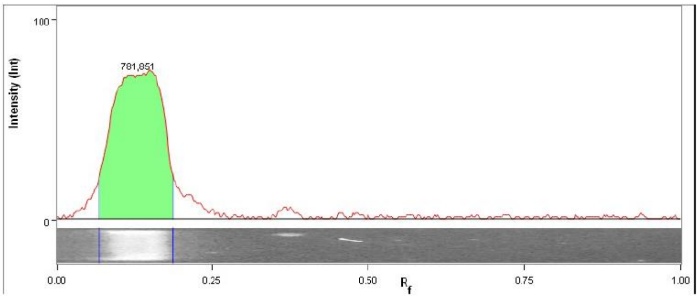

L4= pDNA + FR + *Mucuna* Leaf Extract

Lane 5

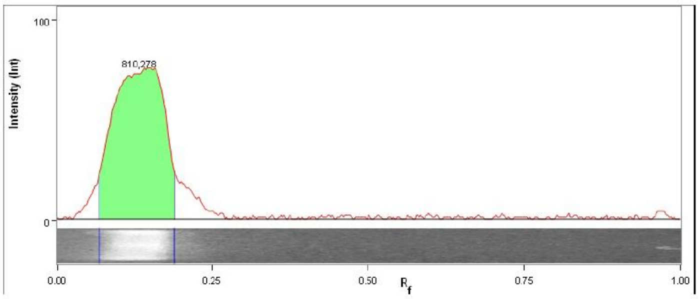

L5= pDNA + FR + *Mucuna* Stem Extract

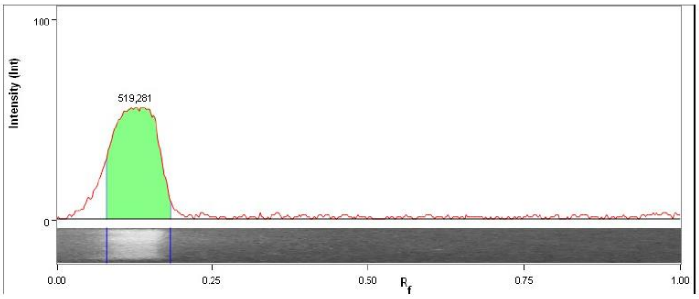

L6= pDNA + FR + *Mucuna* Root Extract

SF4. Pearson's correlation between antioxidant assays

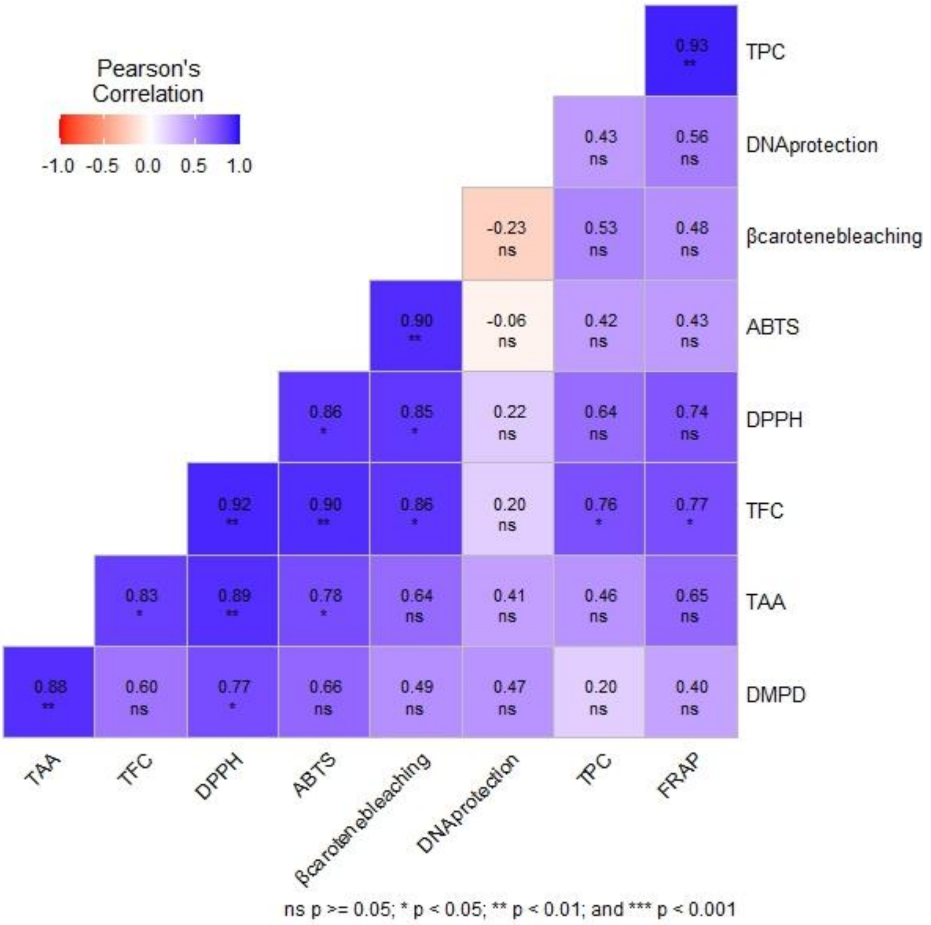

Set up procedure for HPLC-MS/MS instrument for the analysis

Agilent 6200 Series TOF and 6500 Series Q-TOF LC/MS System (Agilent Technology, United States), available in the sophisticated analytical instrument facility (SAIF) of Indian institute of Technology, Bombay was used for high-performance liquid chromatography (HPLC) and tandem mass spectrometry (MS/MS). Full scan and fragment mass was obtained by Time-of-flight (TOF) analyser and chromatographic separation by Hypersil GOLD C18 analytical column (100 x 2.1mm, 3 micron packing) (Thermo Fisher Scientific, United States) at 40ºC. The mobile phase comprised of (A) 0.1% formic acid in water and (B) 90% acetonitrile +10% water+ 0.1% formic acid and applied in the following gradients, 1min – 95% A and 5% B, 20min – 100% B, 25min – 100% B, 26 min – 95% A and 5% B, 30min - 95% A and 5% B. The flow rate and pressure was 0.3mL/min and 1200 bar respectively, injection volume was 5µL and UV spectra was recorded between 190 nm to 400 nm. The LC elute was introduced into electrospray ion source (ESI) and was analyzed in duel positive and negative ion modes at following settings, mass range (m/z) – 125 to 1200, capillary voltage – 3500 V, nozzle voltage – 1000 V, nebulizer pressure – 35 psig, gas flow rate – 13 L/min, gas temperature – 250 ºC. The generated data was analyzed with Agilent MassHunter Workstation Software.

Compound classification

Compound detected by the HRLC-MS/MS analysis were classified into their chemical classes using online tool for chemical taxonomy, classyfire (http://classyfire.wishartlab.com) developed by Feunang et al. (Djoumbou Feunang et al., 2016).

Chromatograms

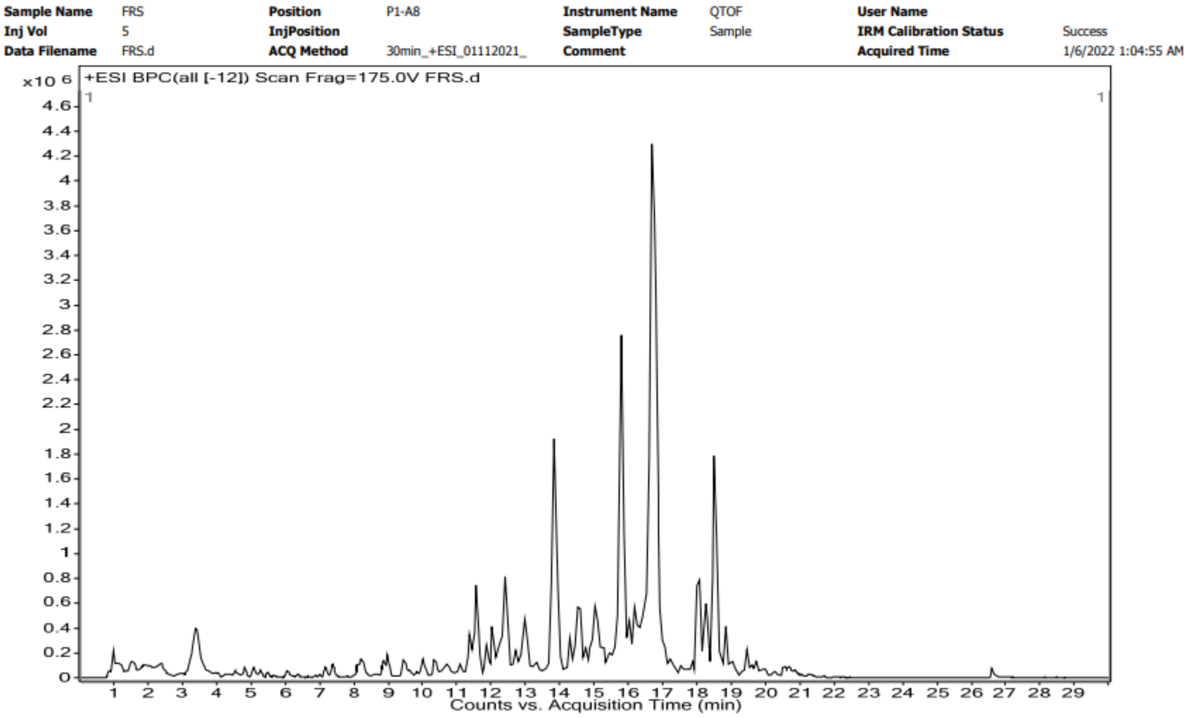

HRLC MS/MS +ESI Chromatogram of *Flemingia* Root

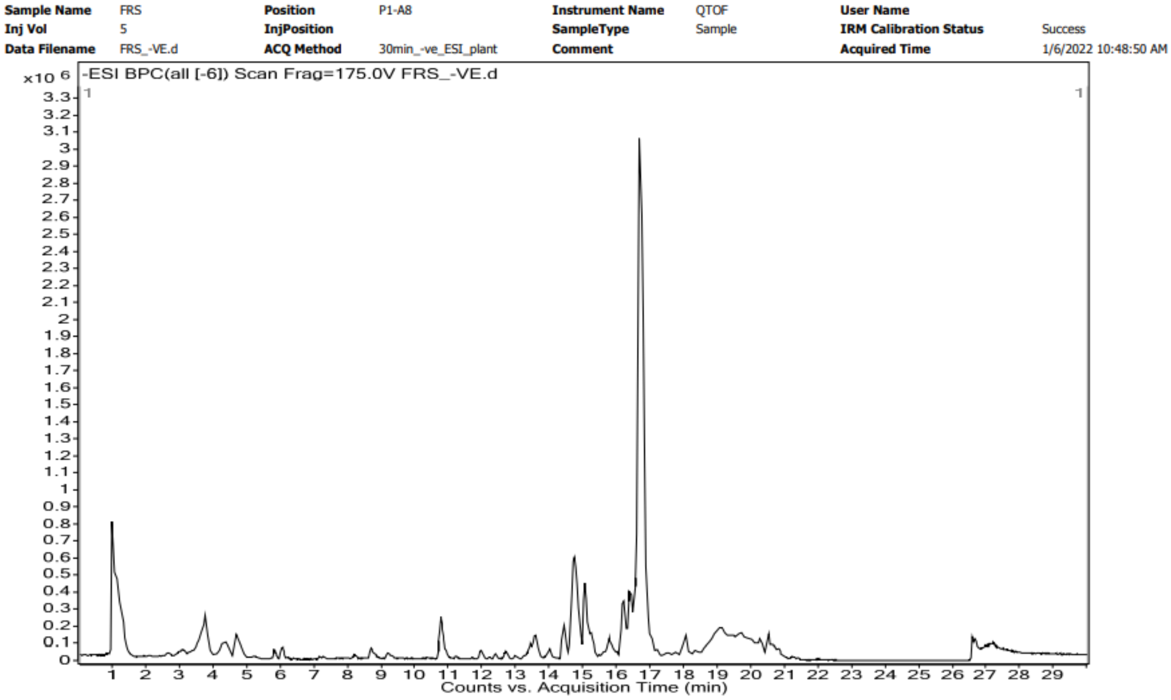

HRLC MS/MS -ESI Chromatogram of *Flemingia* Root

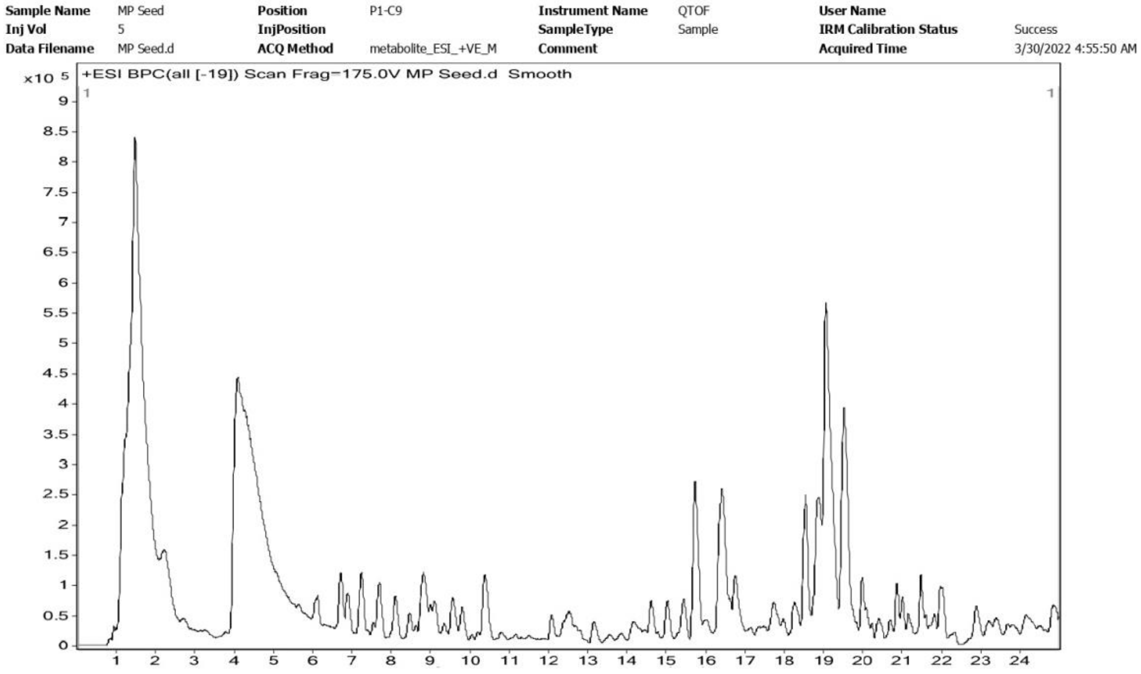

HRLC-MS/MS +ESI Chromatogram of *Mucuna* Seed

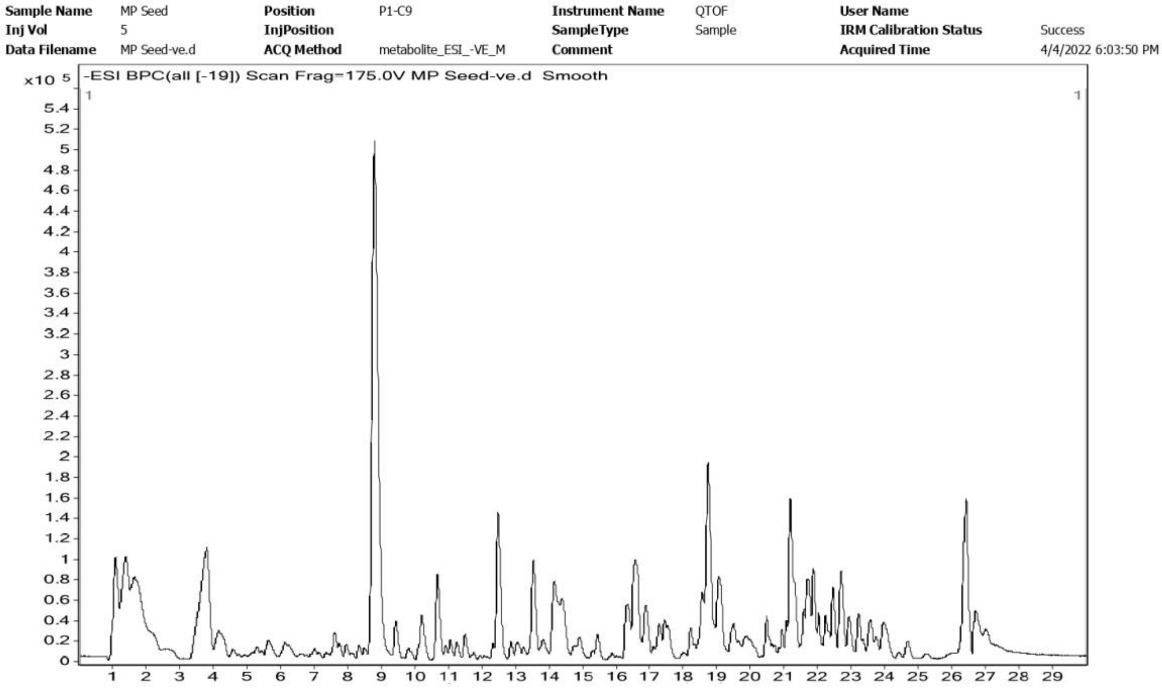

HRLC-MS/MS -ESI Chromatogram of *Mucuna* Seed

Bibliography

Djoumbou Feunang, Y., Eisner, R., Knox, C., Chepelev, L., Hastings, J., Owen, G., Fahy, E., Steinbeck, C., Subramanian, S., Bolton, E., Greiner, R., & Wishart, D. S. (2016). ClassyFire: automated chemical classification with a comprehensive, computable taxonomy. *Journal of Cheminformatics, 8*(1), 61. https://doi.org/10.1186/s13321-016-0174-y
